# Contrasting Spatial Genomic Structure in Two Widespread Grassland Species Across Norway Supports Species‐Specific Seed Sourcing

**DOI:** 10.1002/ece3.74474

**Published:** 2026-11-05

**Authors:** Abel Gizaw, Hans Martin Hanslin, Trygve S. Aamlid, Håkon Borch, Hana Šípková, Carl Gunnar Fossdal

**Affiliations:** ^1^ Division of Environment and Natural Resources, Department of Urban Greening and Vegetation Ecology Norwegian Institute of Bioeconomy Research Ås Norway; ^2^ Department of Botany, Faculty of Science Charles University Prague Czech Republic; ^3^ Department of Ecology, Faculty of Environmental Sciences Czech University of Life Sciences Prague Prague Czech Republic; ^4^ Division of Plant Health and Biotechnology, Department of Molecular Plant Biology Norwegian Institute of Bioeconomy Research Ås Norway

**Keywords:** *Achillea millefolium*, ecological restoration, Norway, population genomics, seed transfer zones, *Silene dioica*

## Abstract

Ecological restoration typically relies on locally sourced seed material, yet seed sourcing in many countries is based on broad administrative regions rather than empirical genetic data. Whether these generalized zones reflect biologically meaningful population genomic structure, particularly among co‐occurring species used together in restoration, remains poorly understood, thus limiting genetically informed restoration practices. Here, we use genome‐wide double‐digest RAD sequencing (ddRAD‐seq) to examine spatial population genomic structure in two widespread perennial grassland species commonly used in restoration in Norway: *Silene dioica* , an obligately outcrossing species with limited seed dispersal, and *Achillea millefolium* , a polyploid complex characterized by high gene flow and dispersal capacity. Analysis of 17 populations of *S. dioica* (355 individuals; 19,200 SNPs) and 23 populations of *A. millefolium* (428 individuals; 21,294 SNPs) across broad latitudinal and climatic gradients in Norway revealed strikingly contrasting patterns of genetic structure. *Silene dioica* showed moderate spatial structure, significant isolation‐by‐distance, and multiple geographically coherent genetic groups, indicating spatially restricted gene flow and supporting the delineation of multiple seed transfer zones. In contrast, *A. millefolium* showed weak population differentiation, extensive admixture, and little geographic structuring, but significant isolation‐by‐environment, with annual precipitation emerging as the strongest environmental predictor of genomic differentiation independently of geographic distance, justifying the delineation of three broad genomic zones. Because species‐specific life‐history traits likely contributed to these patterns, a single generalized seed‐sourcing framework can be inadequate even for sympatric species restored together. While the structured genomic patterns in *S. dioica* support localized seed sourcing, the high connectivity in *A. millefolium* supports broader transfer zones. Overall, our study highlights the importance of incorporating genomic data into restoration planning by aligning seed transfer distances with species‐specific connectivity to reduce genetic risks and maintain evolutionary potential under environmental change.

## Graphical abstract

Seed sourcing for ecological restoration often relies on broad administrative zones that may not reflect underlying genetic structure. Using genome‐wide ddRAD‐seq data from two co‐occurring Norwegian grassland species, we show contrasting patterns: *Silene dioica* exhibits moderate spatial genetic structure indicating the need for localized sourcing, whereas *Achillea millefolium* shows high connectivity across large spatial scales driven instead by precipitation gradients that are consistent with broader transfer zones. These results demonstrate that generalized seed sourcing frameworks are inadequate and highlight the need for species‐specific, genomically informed strategies.

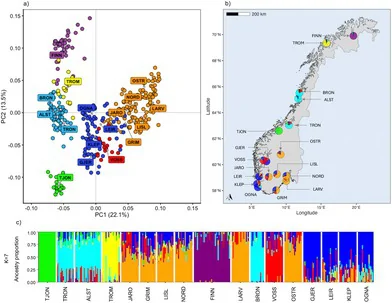

## 1 Introduction

Flowering herbaceous plants form key components of grassland ecosystems, contributing greatly to plant diversity, sustaining pollinator communities, and providing associated ecosystem services (Newbold et al. 2016; Piqueray et al. 2019). However, grasslands are declining globally and are undergoing rapid degradation driven by land‐use and climate change, resulting in substantial loss of biodiversity and ecosystem functioning (Newbold et al. 2015; IPBES 2019). Ecological restoration has therefore become an important strategy for mitigating these impacts and promoting the recovery of resilient grassland ecosystems worldwide. Yet restoration outcomes remain highly variable, reflecting in part the biological and genetic suitability of the seed material used for restoration, making seed sourcing strategies fundamental to long‐term restoration success.

Seed provenance (seed source) greatly influences the demographic and evolutionary outcomes of restoration efforts (McKay et al. 2005; Breed et al. 2018). The genetic composition of plant material strongly shapes establishment success, long‐term persistence, and evolutionary potential. Successful restoration therefore requires balancing the maintenance of genetic diversity and adaptive potential against the risks associated with transferring genetically incompatible plant material. The use of genetically uncharacterized or non‐local seed sources may therefore introduce genotypes poorly matched to local environmental conditions, disrupt locally adapted gene pools and, in extreme cases, reduce fitness through outbreeding depression (Hufford and Mazer 2003; Edmands 2007). However, recent evidence indicates that outbreeding depression within plant species is generally uncommon except where populations are highly divergent, reproductively isolated, or differ in ploidy, whereas reduced genetic diversity and inbreeding often represent more immediate risks for restoration success (Frankham et al. 2011; Bucharova et al. 2019). Although such genetic effects may remain undetected during early recruitment, they can have negative consequences for the resilience and adaptive potential of the established populations across subsequent generations (Frankham et al. 2011). Restoration efforts that do not explicitly incorporate genetic information into seed sourcing strategies may therefore increase the risk of both short‐term failure and long‐term evolutionary decline. To address these risks, genetically informed species‐specific seed transfer zones, geographically defined zones within which seed movement is considered to minimize maladaptation, have been proposed as a crucial component of sustainable restoration practice (Krauss et al. 2013; Bower et al. 2014).

In many regions, seed‐sourcing guidelines are defined according to administrative borders, agroecological zones, or broad climatic regions rather than empirical genetic data (McKay et al. 2005; Broadhurst et al. 2008; Breed et al. 2019; Cevallos et al. 2020). Although such approaches offer logistical simplicity, they may fail to reflect the spatial scale at which local adaptation and population genetic structure are organized. Moreover, this approach assumes that geographic or environmental boundaries correspond to population genetic structure and even that co‐occurring species respond similarly to environmental variables. However, these assumptions are often unfounded and need to be tested empirically. Population genetic structure is shaped by species‐specific combinations of differences in life‐history traits, demographic history, mating system, and dispersal mechanisms that lead to genetic variation in spatial pattern even among sympatric species (Lowe et al. 2017; Bucharova et al. 2019; Durka et al. 2025). Generalized seed transfer zones, as a result, may underestimate the genetic differentiation in genetically structured species while at the same time unnecessarily limiting the movement of seeds in species that are highly connected with gene flow.

In Norway, native regional seed mixtures are increasingly used in grassland restoration and in the establishment of species‐rich meadows. The Norwegian Nature Diversity Act requires practitioners to use seed mixtures of local provenance (Naturmangfoldloven 2009), yet there are no formal guidelines to define what constitutes “local”, both in genetic and geographical sense, creating uncertainty in operational seed sourcing (Jørgensen et al. 2016). However, current seed sourcing strategies for wildflower seed mixtures remain mostly based on 10 administrative regions (Figure 1) rather than on empirical assessment of genetic differentiation and adaptive variation within wild plant populations (NIBIO 2026). Whether such generalized divisions adequately capture biologically meaningful patterns of population structure or adaptive variation across multiple co‐occurring grassland species therefore remains unclear. Addressing this knowledge gap is essential for the long‐term success of restoration efforts as effective outcomes depend on genetically informed species‐specific seed sourcing strategies that form the backbone of regional seed mixtures.

**FIGURE 1:**
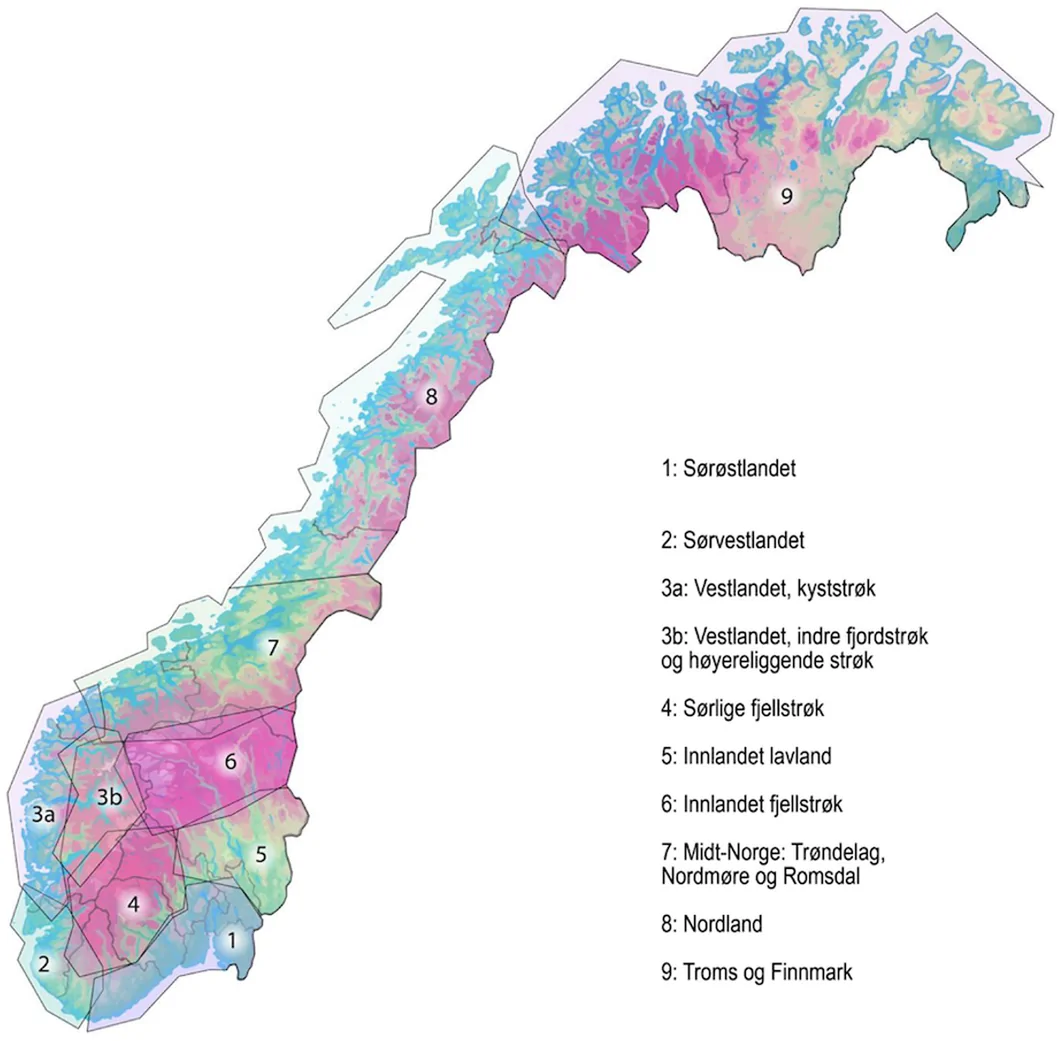
Map showing the current administrative seed‐sourcing regions for native meadow species in Norway. Black polygons delineate the 10 regional seed‐sourcing zones. Elevation is represented by a continuous color gradient, ranging from light green in lowland areas to dark pink at the highest elevations.

Advances in population genomics provide powerful tools to guide seed sourcing strategies. Genome‐wide markers enable robust inference of population connectivity, differentiation, and isolation‐by‐distance (IBD) across broad spatial scales, offering a quantitative basis for identifying biologically meaningful seed sourcing units (e.g., Jørgensen et al. 2016; Fremout 2021; Durka et al. 2025). Reduced‐representation sequencing approaches such as double‐digest RAD sequencing (ddRAD‐seq) are particularly well suited for non‐model plant species commonly used in restoration, allowing thousands of loci to be surveyed across large numbers of populations at manageable cost. Although neutral genomic structure does not quantify local adaptation, it provides essential insight into evolutionary independence and gene flow, which are fundamental to assessing genetic risks associated with seed transfer (Waples and Gaggiotti 2006; Breed et al. 2019).

Here, we use genome‐wide ddRAD‐seq to infer spatial population genomic structure in two widely used perennial wildflower species commonly used in regional seed mixtures in Norway: *Silene dioica* (Caryophyllaceae) and *Achillea millefolium* (Asteraceae). Both species are broadly distributed across Europe, and frequently used together in ecological restoration, yet they differ markedly in reproductive system and dispersal mechanisms. *Silene dioica* is a dioecious, diploid, and self‐incompatible species relying on insect‐mediated pollen transfer and lacking mechanisms for long‐distance seed dispersal (Šmarda et al. 2019). Such traits are typically associated with restricted effective gene flow, strong spatial genetic structure, and isolation by distance (Loveless and Hamrick 1984; Hamrick and Godt 1996; Barrett 2002; Duminil et al. 2007). Moreover, in northern Europe, postglacial recolonizations from multiple refugia have contributed to regional genetic differentiation in many plant species (Taberlet et al. 1998; Alsos et al. 2005; Eidesen et al. 2007; Prentice et al. 2008), suggesting both historical and contemporary processes may shape the genetic structure within species.

In contrast, *A. millefolium* represents a widespread polyploid species complex characterized by broad ecological tolerance. Across Europe, ploidy levels vary from diploid (2n = 18) to octoploid (2n = 72), reflecting an ability to colonize varied environments (Pustahija et al. 2013; Lopez‐Vinyallonga et al. 2015). However, the Northern European lineages, particularly those from Norway, are known to be more uniform, with chromosome counts confirming a stable hexaploid cytotype (2n = 6× = 54; Baltisberger and Widmer 2016). The species exhibits generalist insect pollination through numerous open flowers, and wind‐dispersed achenes that facilitate long‐distance dispersal. While high dispersal capacity promotes extensive gene flow across landscapes, polyploidy may buffer populations against genetic drift and reduce the fitness costs associated with admixture (Soltis and Soltis 2000; Parisod et al. 2010; Meirmans et al. 2018). European populations of *A. millefolium* show weak geographic structure and low population differentiation, consistent with recent expansion and ongoing connectivity (Hörandl and Paun 2007; Guo et al. 2012; Lopez‐Vinyallonga et al. 2015).

Based on these contrasting biological and evolutionary characteristics, we predicted that the obligate outcrossing and limited seed dispersal of *S. dioica* would generate relatively moderate isolation by distance, moderate population differentiation, and lower levels of admixture. In contrast, the wind‐dispersed and polyploid *A. millefolium* would exhibit lower genetic differentiation, weaker isolation by distance, and more continuous genetic variation with higher levels of admixture. To evaluate these predictions and their implications for restoration practice, we address three specific questions: (1) Do *S. dioica* and *A. millefolium* differ in the magnitude and spatial patterns of genomic structure across Norway? (2) Do inferred patterns of genetic structure align with the current use of administratively defined seed‐sourcing regions, or do they instead support species‐specific seed transfer zones? And (3) How can genome‐wide population genomic data be translated into practical seed‐sourcing zones that identify genetically compatible seed transfer units rather than prioritizing individual source populations?

## 2 Materials and Methods

### 2.1 Plant Material and Sampling

#### 2.1.1 Studied Species

This study examines two native and widely distributed wildflower species that are commonly used together in seed mixtures for restoration in Norway: *Achillea millefolium* L. (Asteraceae) and *Silene dioica* (L.) Clairv. (Caryophyllaceae). Both species are among the most widely used components of regional seed mixtures in Norway. Their adaptability to wide climatic conditions and their importance in attracting a wide variety of pollinators make them ideal candidate species for assessing spatial genetic structuring relevant to seed sourcing for restoration.

#### 2.1.2 Sampling Strategies

Leaf sampling was conducted across a wide geographic and climatic gradient in Norway, covering the natural distributional range of both species. Fresh leaf material was collected from several populations, either directly in the field or from cultivated plants. All cultivated plants were raised from wild‐collected seeds originating from known source populations. To the best of our knowledge, all sampling localities represent naturally occurring populations with no history of seeding or restoration activities. Prior to sowing, seeds from multiple individuals were thoroughly mixed to minimize overrepresentation of any single maternal line. Our sampling strategy in the field involved collecting leaf samples, wherever possible, from approximately 20 individuals per population, with each sampled individual separated by ~10–15 m apart. Leaf samples were immediately dried in silica gel to preserve DNA integrity. In total, we sampled 17 populations comprising 366 individuals of *S. dioica* and 23 populations comprising 455 individuals of *A.millefolium* . Details regarding population sizes, number of samples per population, sampling locations, geographic coordinates, and corresponding administrative seed‐sourcing regions for each population are provided in Table 1.

**TABLE 1:** Sampling locations, altitude, geographic coordinates, sample size, current seed‐sourcing region, and proposed genomic zone for *S. dioica* and *A. millefolium* populations included in the study based on ddRAD‐seq data. Population identifier (PopID), locality names, altitude in meter (Alt), latitude (Lat.), and longitude (Long.) and the number of sampled individuals per population (*n*) are provided.

PopID	Locality	Alt (m)	Lat.	Long.	*n*	Current seed‐sourcing region	Proposed genomic zone
*Silene dioica*							
GRIM	Grimstad, Holvika	10	58.341	8.523	20	Region 1: Sørøstlandet	Southeastern inland zone
LARV	Larvik, Larvik	57	59.058	10.024	20	Region 1: Sørøstlandet	Southeastern inland zone
NORD	Nordbylendet, Larvik	124	59.458	10.031	20	Region 1: Sørøstlandet	Southeastern inland zone
KLEP	Klepp, Rogaland	44	58.774	5.639	20	Region 2: Sørvestlandet	Southwestern coastal zone
OGNA	Ogna, Hå	16	58.508	5.803	18	Region 2: Sørvestlandet	Southwestern coastal zone
GJER	Gjerdet, Ulvik	120	60.561	6.896	20	Region 3b: Vestlandet	Southwestern coastal zone
LEIR	Leirå, Nedstrand, Tysvær	15	59.343	5.877	18	Region 3b: Vestlandet	Southwestern coastal zone
VOSS	Voss	61	60.627	6.434	20	Region 3b: Vestlandet	Southwestern coastal zone
JARO	Lii i Jåro, Bykle	680	59.355	7.346	20	Region 4: Sørlige fjellstrøk	Southeastern inland zone
LISL	Lislero, Hjartdal	409	59.590	8.590	20	Region 4: Sørlige fjellstrøk	Southeastern inland zone
OSTR	Østre Slidre	855	61.123	9.121	20	Region 6: Innlandet fjellstrøk	Southeastern inland zone
TJON	Tjønneberget, Surnadal	24	62.974	8.780	20	Region 7: Midt‐Norge	TJON‐local zone
TRON	Trondheim Væresholmen	102	63.419	10.493	20	Region 7: Midt‐Norge	Central Norway zone
ALST	Alstahaug	560	65.924	12.500	30	Region 8: Nordland	Central Norway zone
BRON	Brønnøy	1	65.466	12.226	20	Region 8: Nordland	Central Norway zone
FINN	Skoganvarre, Finnmark	124	69.830	25.101	40	Region 9: Troms og Finnmark	Eastern Arctic zone
TROM	Tromsø	23	69.654	18.912	20	Region 9: Troms og Finnmark	Western Arctic zone
*Achillea millefolium*							
DaeLI	Dæliveien, Bærum	127	59.915	10.524	20	Region 1: Sørøstlandet	South‐central eastern zone
GJEV	Gjevinggården, Tvedestrand	20	58.651	9.120	20	Region 1: Sørøstlandet	South‐central eastern zone
GRIM	Grimstad	10	58.341	8.523	20	Region 1: Sørøstlandet	South‐central eastern zone
KLEP	Klepp, Ølbergskogen	44	58.774	5.639	20	Region 2: Sørvestlandet	Western coastal zone
LIST	Lista, Farsund	6	58.067	6.796	20	Region 2: Sørvestlandet	Western coastal zone
OGNA	Ogna, Hå	10	58.508	5.799	20	Region 2: Sørvestlandet	Western coastal zone
FORD	Klokkarvik, Førde	60	60.628	6.434	20	Region 3b: Vestlandet	Western coastal zone
KJER	Kjeringvika, Nedstrand, Tysvær	3	59.341	5.881	19	Region 3b: Vestlandet	Western coastal zone
VAKS	Vaksdal, Vestland	310	60.591	5.835	20	Region 3b: Vestlandet	Western coastal zone
VALU	Valufsin, Seljord	515	59.590	8.583	20	Region 4: Sørlige fjellstrøk	VALU‐local zone
ABJO	Åbjør grendehus, Valdres	483	60.930	9.307	20	Region 6: Innlandet fjellstrøk	South‐central eastern zone
ASEB	Åsebygd, Valdres	504	60.726	9.691	20	Region 6: Innlandet fjellstrøk	South‐central eastern zone
OSSL	Øystre Slidre	547	61.141	9.085	20	Region 6: Innlandet fjellstrøk	South‐central eastern zone
OSTR	Øystre Slidre	800	61.123	9.121	20	Region 6: Innlandet fjellstrøk	South‐central eastern zone
AKSN	Aksneset, Tingvoll	11	62.957	8.331	18	Region 7: Midt‐Norge	Western coastal zone
MORE	Gjøra, Sunndal	500	62.521	9.082	20	Region 7: Midt‐Norge	South‐central eastern zone
STOR	Storlia, Indre Fosen	270	63.693	10.521	20	Region 7: Midt‐Norge	South‐central eastern zone
TRON	Trondheim Væresholmen	102	63.419	10.493	20	Region 7: Midt‐Norge	Western coastal zone
ALST	Alstahaug	562	65.924	12.500	19	Region 8: Nordland	ALST‐local zone
TOMM	Tømmervika, Alstashaug	580	66.924	12.500	20	Region 8: Nordland	South‐central eastern zone
ANDS	Andselv, Målselv	180	69.072	18.443	19	Region 9: Troms og Finnmark	Northern zone
LANE	Lanesbogen, Senia	34	69.341	17.927	20	Region 9: Troms og Finnmark	Northern zone
TROM	Tromsø, Senja	24	69.654	18.912	20	Region 9: Troms og Finnmark	Northern zone

*Note:* Region 3b: Vestlandet = Region 3b: Vestlandet (indre fjordstrøk og høyereliggende strøk); Region 7: Midt‐Norge = Region 7: Midt‐Norge: Trøndelag, Nordmøre og Romsdal.

#### 2.1.3 Flow Cytometry (FCM)

To confirm ploidy level in both species, a subset of individuals from ddRAD dataset (35 *S. dioica* from seven populations and 80 *A. millefolium* from 14 populations) was screened using flow cytometry (Partec CyFlow ML; 375 nm UV LED; DAPI) at the Suda Laboratory of Flow Cytometry, Charles University, following established protocols (Suda and Trávníček 2006; Doležel et al. 2007). All screened individuals were uniformly diploid ( *S. dioica* ) or hexaploid ( *A. millefolium* ), showing no evidence of mixed‐ploidy populations in either species.

#### 2.1.4 DNA Extraction and ddRAD‐Seq

Genomic DNA was extracted from 10 to 15 mg of silica dried leaf tissue, which was ground to a fine powder using TissueLyser (QIAGEN TissueLyser II). Genomic DNA was extracted with the beadex Livestock kit (LGC Biosearch Technologies) using the BioSprint automated extraction platform (QIAGEN), following the manufacturer's instructions. The extracted DNA was eluted in 70 μL of the elution buffer and its concentration was measured using Qubit fluorometer (Invitrogen by Life technologies). All DNA extractions were performed at BioBank AS (Norway).

Approximately 200 ng of extracted high‐quality genomic DNA for each sample was sent to BGI Genomics (Beijing, China) for ddRAD library preparation. Libraries were prepared following the protocol of Peterson et al. (2012) using the following brief modifications: DNA concentrations were normalized and digested using *Eco*RI and *Mse*I enzymes, followed by adapter ligation. Library quality and fragment size distribution in the 350–550 bp size range were assessed using a Qubit Fluorometer and Fragment Analyzer prior to sequencing. The final ddRAD libraries were sequenced as multiplexed libraries on shared lanes of the DNBSEQ G‐400 platform using 150 bp paired‐end reads, producing an average of approximately 1.9 Gb of clean sequence data per sample.

#### 2.1.5 SNP Processing and Filtering

Raw sequence data were processed at BGI Genomics, where reads were demultiplexed by barcode and quality‐filtered to remove low‐quality bases, adapter sequences, and reads with ambiguous barcodes. Cleaned reads were then analyzed using BGI's standard de novo assembly and diploid genotype‐calling pipeline, generating variant call format (VCF) files for subsequent filtering and downstream and population genomic analyses.

Because *A. millefolium* is known to exhibit multiple ploidy levels across Europe (Lopez‐Vinyallonga et al. 2015), and accurate allele dosage estimation is challenging in ddRAD‐seq datasets due to limited read‐depth, genotypes were analyzed under a diploid framework for both species to ensure methodological consistency and enable direct comparison across species (Blischak et al. 2018; Meirmans et al. 2018; Durka et al. 2025). Downstream variant filtering and data preparation were conducted using BCFtools v1.21 (Danecek et al. 2021), VCFtools v0.1.16 (Danecek et al. 2011), and PLINK v2.0 (Chang et al. 2015). VCF files were first filtered to remove insertions and deletions using BCFtools, followed by filtering with VCFtools to retain high‐quality, biallelic SNPs for further analyses.

We employed two separate filtering approaches: one optimized for the estimation of genetic diversity metrics and a second designed for downstream population genomic analyses. For genetic diversity estimation, including observed and expected heterozygosity (*H*_*E*_, *H*_*O*_), nucleotide diversity (π), and inbreeding coefficient (*F*_*IS*_), we used a minimally filtered, locus‐level dataset to avoid bias associated with overly stringent filtering (O'Leary et al. 2018; Linck and Battey 2019). Specifically, locus‐level filtering was applied, retaining SNPs with a minimum minor allele frequency (MAF) ≥ 0.01 and ≤ 10% missing genotypes per locus, and excluding loci that deviated significantly from Hardy–Weinberg equilibrium (*p* < 1 × 10^−6^). To reduce linkage disequilibrium among closely spaced loci, SNP thinning was applied by retaining a single SNP per kilobase.

For downstream population genomic analyses, including PCA and population structure inferences, we applied more stringent filtering to reduce non‐independence among loci and improve the inference robustness (Patterson et al. 2006). This included retaining SNPs with MAF ≥ 0.05, genotype depth ≥ 3 to remove low‐confidence genotype calls, ≤ 25% missing data per site, and HWE *p* values > 1 × 10^−15^. The resulting dataset was subsequently filtered using a minimum mean depth threshold of DP ≥ 10 and thinned to one SNP per kilobase, and SNPs and individuals with > 10% and > 15% missing data were removed, respectively, to produce the final PLINK‐formatted dataset used for structure and differentiation analyses.

#### 2.1.6 Population Genomic Analysis

Population genomic analyses were performed using a combination of R v4.3.1 (R Core Team 2023) and other specific tools, as detailed below. We first examined patterns of genetic variation among individual samples using principal component analysis (PCA) implemented in PLINK v1.9 (Purcell et al. 2007). The resulting PCA eigenvectors were plotted and visualized in R using the *ggplot2* package v. 3.5.1 (Wickham 2016).

Population genetic structure was assessed using the model‐based Bayesian clustering algorithm implemented in ADMIXTURE v1.3 (Alexander et al. 2009). ADMIXTURE estimates individual ancestry proportions and assigns individuals to a specified number of ancestral populations based on a maximum‐likelihood framework and with no prior information on population origin. Analyses were conducted for *K* values ranging from 2 to 10, with multiple independent runs at every *K* using different random seeds to assess convergence of inferred clusters. The most likely number of ancestral populations was determined by identifying the value of *K* with the lowest cross‐validation (CV) error and further evaluated for biological plausibility. ADMIXTURE *Q*‐matrix outputs, together with sampling coordinates, were used in the R package *mapmixture* v1.2.0 (Jenkins 2024) to generate both STRUCTURE‐style bar plots and spatial admixture maps. Spatial patterns of ancestry were further visualized by plotting site‐level ancestry proportions as pie charts overlaid on a high‐resolution Norway shapefile obtained from *geoBoundaries* v.6.0.0 (Runfola et al. 2020; CC BY 4.0 license). Sampling locations were assessed relative to the current seed‐sourcing regions to facilitate comparison between the existing operational framework and the inferred genomic structure identified in this study.

Genetic diversity within populations was assessed by estimating individual‐level observed and expected heterozygosity (*H*_*O*_, *H*_*E*_) and Wright's inbreeding coefficient (*F*_*IS*_) using VCFtools v0.1.16 via the ‐‐*het* function (Danecek et al. 2011). Population‐level heterozygosity and *F*_*IS*_ were additionally calculated in PLINK v1.9 using a population assignment file (‐‐*within*), with summary statistics generated in R. Nucleotide diversity (π) was estimated for each population by sub‐setting the filtered VCF to the corresponding individuals and computing site‐wise π (‐‐*site‐pi*) in VCFtools. Site‐wise values were subsequently averaged across loci to obtain population‐level estimates. Pairwise genetic differentiation among populations was estimated using Weir & Cockerham unbiased *F*_*ST*_ estimator (Weir and Cockerham 1984) implemented in PLINK v 2.0 (Chang et al. 2015). For each pair, mean and weighted *F*_*ST*_ values were extracted from the PLINK log files and assembled into a symmetric population × population matrix. This weighted *F*_*ST*_ matrix was then visualized using the *preheatmap* R package (Kolde 2025).

Isolation‐by‐distance (IBD) and isolation‐by‐environment (IBE) were examined independently for both species to assess the relationships of geographic and environmental factors with patterns of genetic differentiation (pairwise *F*_*ST*_) at the population level. Geographic distance was calculated as great‐circle (Haversine) distance, using the R package *geosphere* (Hijmans et al. 2017). Environmental distance matrices were calculated from elevation (DEM) and bioclimatic variables from the CHELSA v2.1 database (Karger et al. 2017). To reduce multicollinearity among environmental predictors, bioclimatic variables were screened using pairwise correlations and variance inflation factors (VIF), retaining four variables: annual mean temperature (BIO1), temperature seasonality (BIO4), annual precipitation (BIO12), and precipitation seasonality (BIO15) for subsequent analyses. Mantel tests (Mantel 1967) with 9999 permutations, as implemented in the R package *vegan* (Oksanen et al. 2017), were used to assess the association between genetic and geographic distance matrices (IBD) and between genetic and environmental distances matrices (IBE). We further examined IBD by fitting a linear regression of *F*_*ST*_ against geographic distance, and the relationship was visualized using *ggplot2*, with scatter plots overlaid by fitting regression lines and two‐dimensional kernel‐density contours. Where significant IBE was detected, environmental drivers of genetic differentiation were further investigated using distance‐based redundancy analysis (dbRDA) in *vegan*. Partial dbRDA conditioned on latitude and longitude was used to account for spatial structure, and model significance and marginal effects were tested using separate 9999 permutations.

## 3 Results

### 3.1 Genomic Data and SNP Filtering

The initial ddRAD‐seq dataset for 366 individuals of *S. dioica* generated 3,186,744 putative SNPs (mean sequencing depth = 8.67×; Table S1). After quality, Hardy–Weinberg, missingness, and linkage‐based filtering, the final dataset comprised 355 individuals following the removal of 11 individuals (3.0%) by the > 15% individual missingness filter, genotyped at 19,200 SNPs (overall genotype call rate = 96.8%). For *A. millefolium* , the initial ddRAD‐seq dataset for 455 individuals generated 8,896,919 putative SNPs (mean sequencing depth = 5.87×; Table S1). After applying equivalent filtering, the final dataset comprised 428 individuals following removal of 27 individuals (5.9%) by the > 15% individual missingness filter, genotyped at 21,294 SNPs (overall genotype call rate = 96.5%). These datasets were used for all downstream population genomics analyses.

### 3.2 Genome‐Wide Population Structure

#### 3.2.1 Principal Component Analysis

The PCA revealed a contrasting pattern of spatial genetic structure between *S. dioica* and *A. millefolium* . In *S. dioica* , the first two PCA axes together explained 35.6% of the total variation (PC1: 22.1%; PC2: 13.5%) and revealed moderate but spatially coherent geographic structuring among Norwegian populations (Figure 2a). Along PC1, eastern and southeastern populations clustered toward the positive axis, whereas western and central populations occupied central positions in the ordination. Most populations from the west coast were centrally positioned along the axis, flanked on the left by populations from the central region and on the right by populations from the eastern and southeastern regions. One population from central Norway (TJON) formed a well‐isolated cluster along the negative end of PC1, clearly separated from all other populations. Along PC2, northern populations showed additional differentiation, with the Finnmark population (FINN) appearing to have formed a well‐distinct divergent cluster at the upper end of the left axis, with a population from Tromsø (TROM) positioned slightly below but partially overlapping.

**FIGURE 2:**
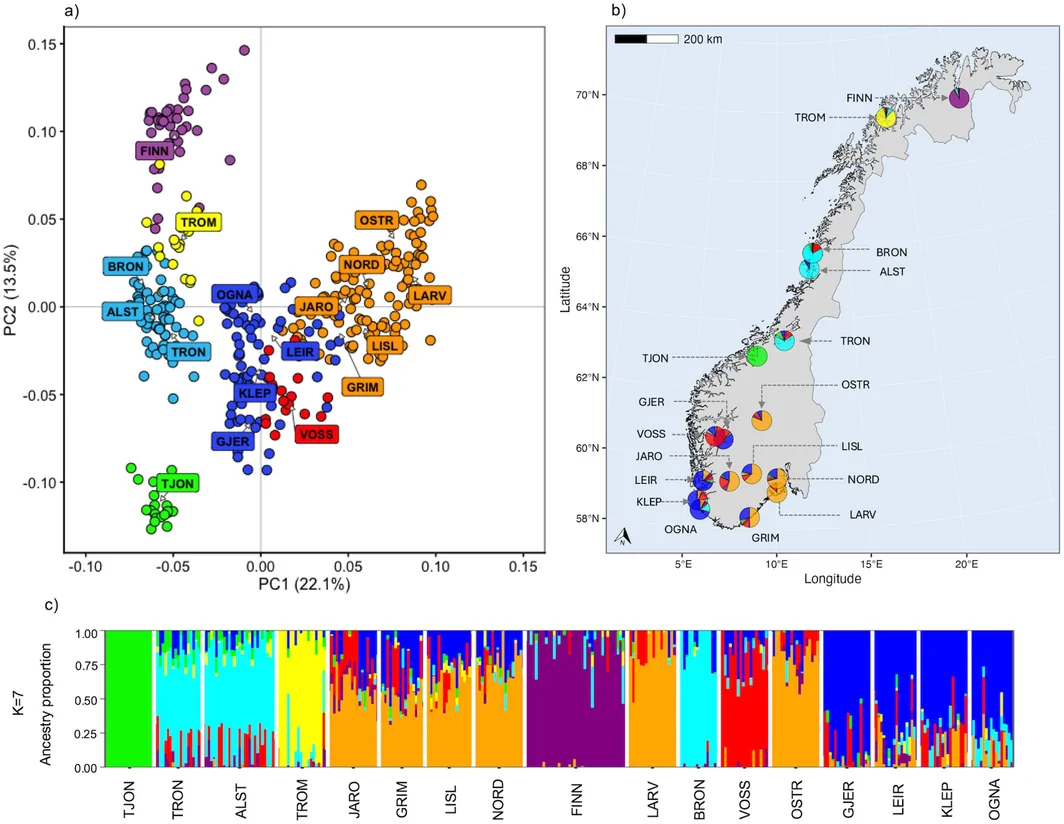
Genetic structure of *S. dioica* across Norway inferred from ddRAD‐seq. (a) Principal component analysis. (b) Spatial visualization of ADMIXTURE ancestry components shown as pie‐charts at each sampled locality, where each circle represents one population positioned according to the sampling coordinates. (c) ADMIXTURE individual ancestry bar plot for the optimal number of clusters (*K* = 7), where each vertical line represents a single individual. Across all panels, colors represent the optimal groups (*K* = 7) inferred. PopID names follow the locality information given in Table 1.

In contrast, *A. millefolium* showed a largely continuous and weak pattern of genetic variation across Norway, with the first two axes explaining 31.4% of the total variation (PC1: 15.9%; PC2: 15.5%; Figure 3a). Western, central, and eastern populations formed a broad overlapping cluster along PC1 with no clear regional separation. Northern populations, including TROM, were positioned toward the positive end of PC1 but remained within the broader cloud of continuous variation. Individuals from ALST formed a slightly separate cluster further up on PC2, but the most noticeable isolated cluster was formed by individuals from VALU, located at the opposite negative end of PC2. Removal of VALU resulted in a similar PCA displaying a weak and continuous pattern of variation, with no clear structure among the remaining populations (Figure S1).

**FIGURE 3:**
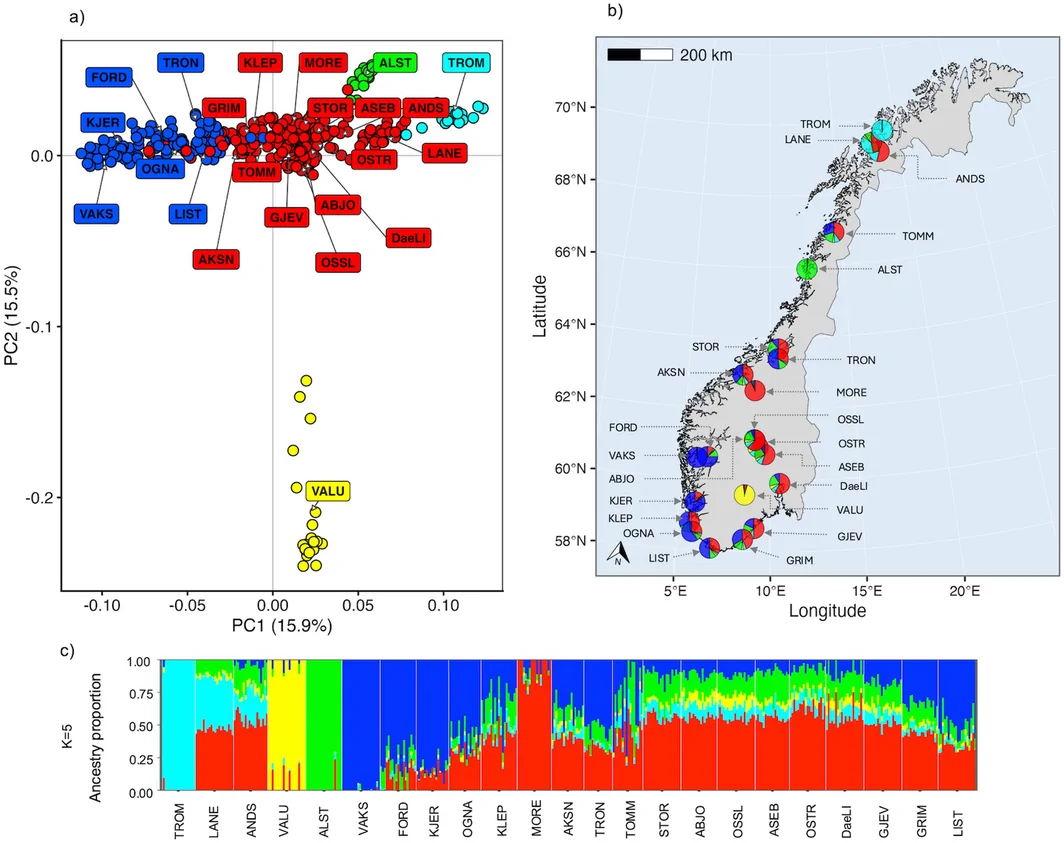
Genetic structure of *A. millefolium* across Norway inferred from ddRAD‐seq. (a) Principal component analysis. (b) Spatial visualization of ADMIXTURE ancestry component shown as pie‐chart at each sampled locality, where each circle represents one population and positioned according to the sampling coordinates. (c) ADMIXTURE individual ancestry bar plot for the optimal number of clusters (*K* = 5), where each vertical line represents a single individual. Across all panels, colors represent the optimal groups (*K* = 5) inferred. PopID names follow the locality information given in Table 1.

#### 3.2.2 Admixture Analysis

In *S. dioica* , admixture analysis showed a steady decrease in CV error from *K* = 2 to *K* = 10 without reaching a clear minimum (Table S2). Therefore, the optimal *K* was determined by evaluating the nature of admixture and the biological coherence of clustering patterns, which identified *K* = 7 as the most informative representation of the population structure, showing strong concordance with the PCA (Figure 2a,b). At *K* = 7, southwestern, southeastern, and central populations formed distinct ancestry groups, while the northern region further subdivided into Tromsø (TROM, Western Arctic) and Finnmark (FINN, Eastern Arctic). The TJON population formed a nearly pure and clearly isolated ancestry cluster with minimal admixture (Figure 2c). ADMIXTURE results for *K* = 2–7 are shown in Figure S3a.

In contrast, admixture patterns of *A. millefolium* showed weak structure and a high degree of admixture, with the CV error declining sharply from *K* = 2 to *K* = 4 and reaching its minimum at *K* = 5 (CV = 0.37459; Table S2), indicating *K* = 5 as the optimal statistically‐supported group (Figure 3b,c). At *K* = 5, most populations exhibited high levels of admixture, and ancestry components were broadly shared across regions, consistent with the continuous genomic structure observed in the PCA (Figure 3a). Nevertheless, the VALU and ALST populations, together with the northern TROM population, showed reduced admixture and were dominated by single ancestry components, while VAKS and MORE also displayed dominant clusters relative to more admixed populations nearby. Importantly, these distinct ancestry components were not geographically exclusive and occurred at lower frequencies in multiple populations (Figure 3b,c), indicating localized allele‐frequency shifts rather than discrete spatial structure. ADMIXTURE results for *K* = 2–5 are shown in Figure S3b.

### 3.3 Genetic Differentiation and Isolation by Distance

Analysis of molecular variance revealed substantial among‐population differentiation among *S. dioica* populations across Norway, with 7.2% of the total genetic variance partitioned among populations (*Φ*_*ST*_ = 0.072, *p* < 0.001; Table 2). Pairwise *F*_*ST*_ estimates indicated moderate to high levels of genetic differentiation, with the highest divergence observed between TJON and OSTR (*F*_*ST*_ = 0.253) and the lowest between ALST and TRON (*F*_*ST*_ = 0.023; mean = 0.130; Figure 4a). In contrast, *A. millefolium* exhibited weak population structure, with only 2.4% of genetic variance attributed to among population differentiation (*Φ*_*ST*_ = 0.024, *p* < 0.001; Table 2). Pairwise *F*_*ST*_ values were generally low, ranging from 0.005 between ABJO and ASEB to 0.105 between TROM and VALU (mean *F*_*ST*_ = 0.041; Figure 4c).

**TABLE 2:** Analysis of molecular variance (AMOVA) partitioning of genome‐wide genetic variation among and within populations of *S. dioica* (19,200 SNPs *n* = 355 individuals) and *A. millefolium* (21,294 SNPs; 428 individuals) based on ddRAD‐seq.

Species	Source of variation	df	SSD	MSD	Variance component (*σ* ^2^)	% of variation	*Φ*_*ST*_	*p*
*S. dioica*	Among populations	16	30.113	1.673	0.0531	7.22	0.072	< 0.001
	Within populations	338	229.207	0.682	0.6822	92.78	—	—
	Total	354	259.320	0.733	—	100	—	—
*A. millefolium*	Among populations	22	21.606	0.982	0.0168	2.45	0.024	< 0.001
	Within populations	405	271.217	0.670	0.6697	97.55	—	—
	Total	427	292.823	0.686	—	100	—	—

*Note:* Variance components (*σ* ^2^), percentage of total variation (%), and fixation index (*Φ*_*ST*_) are shown for each hierarchical level.

**FIGURE 4:**
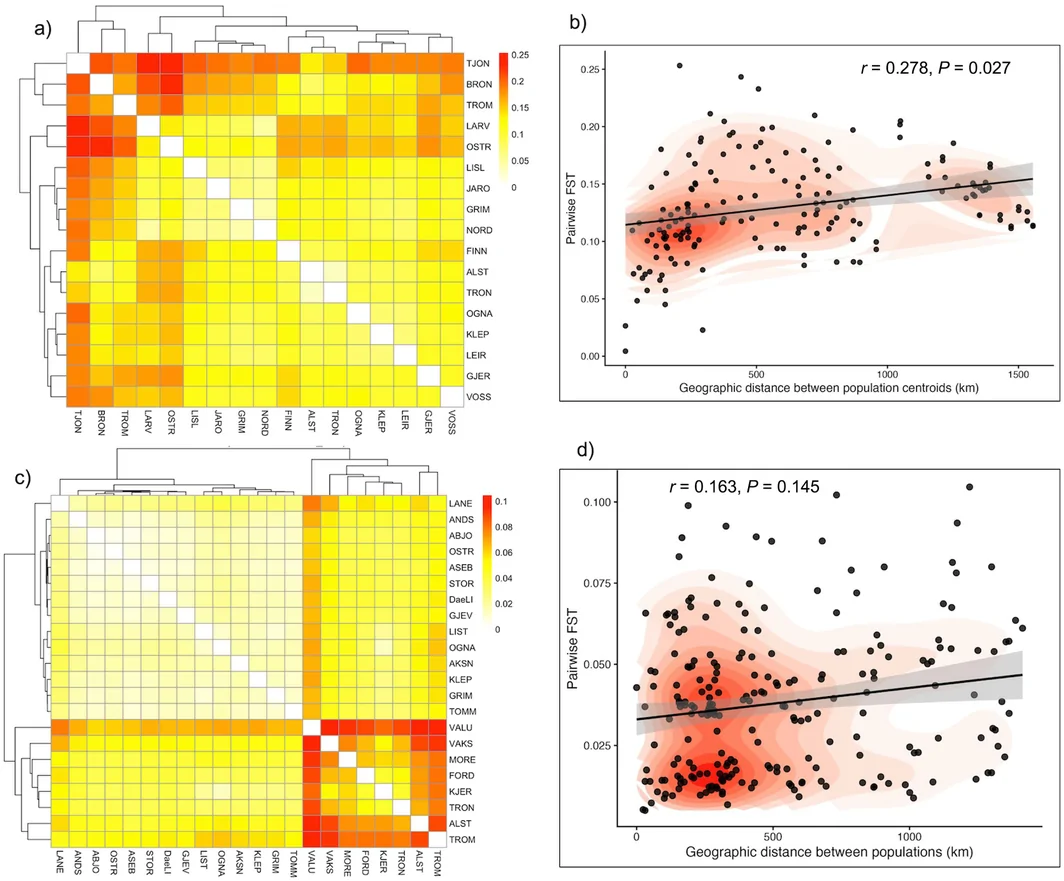
Population genetic differentiation (*F*_*ST*_) and spatial structure in *S. dioica* and *A. millefolium* . Heatmaps show pairwise genetic differentiation among populations for *S. dioica* (a) and *A. millefolium* (c), with hierarchical clustering based on genetic distances. Corresponding relationships between genetic differentiation and geographic distance are shown in (b) and (d), respectively, showing patterns of IBD.

Isolation‐by‐distance analysis revealed a significant positive relationship between genetic and geographic distance in *S. dioica* (Mantel test: *r* = 0.278, *p* = 0.027). Pairwise comparisons were dominated by intermediate levels of genetic differentiation (*F*_*ST*_ = 0.100–0.150), with increasing density at greater geographic distance (Figure 4b). Linear regression analysis showed a significant IBD pattern (*R* ^*2*^ = 0.077, *p* < 0.001; Table S3). A separate regression analysis based on Rousset's approach further supported a strong relationship, with genetic differentiation markedly increasing with log‐transformed geographic distance (*R* ^2^ = 0.180, *p* = 5.6 × 10^−9^, Figure S2a). By comparison, *A. millefolium* displayed weak spatial genetic structuring with no significant correlation between genetic and geographic distance based on the Mantel test (*r* = 0.163, *p* = 0.145; Figure 4d). Although linear regression analyses showed a significant but weak increase in *F*_*ST*_ with geographic distance (*p* = 0.013), the strength of the relationship was low (*R* ^2^ = 0.027, *p* = 0.013; Table S3). Pairwise *F*_*ST*_ values showed little change across distances, clustering at low levels of differentiation (*F*_*ST*_ = 0.020–0.060), and only a slight increase in density at greater distances (Figure 4d). Regression analysis based on Rousset's a similarly revealed a weak association with log‐transformed geographic distance (*R* ^2^ = 0.021, *p* = 0.029; Table S3). In contrast to *S. dioica* , environmental distance was significantly associated with genetic differentiation in *A. millefolium* (Mantel test: *r* = 0.276, *p* = 0.024; Figure 5a). Distance‐based redundancy analysis further demonstrated a significant effect of environmental variation on genomic differentiation (overall dbRDA: *F* = 3.33, *p* < 0.001; Figure 5b), which remained significant after controlling for geographic location (partial dbRDA: *F* = 3.27, *p* < 0.001). Marginal permutation tests of the partial dbRDA identified annual precipitation (BIO12) as the only environmental variable that explained a significant proportion of genetic differentiation after controlling for geographic effects (*F* = 2.46, *p* = 0.0477; Figure 5b). Annual mean temperature, temperature seasonality, precipitation seasonality, and elevation were not significant (Table S5).

**FIGURE 5:**
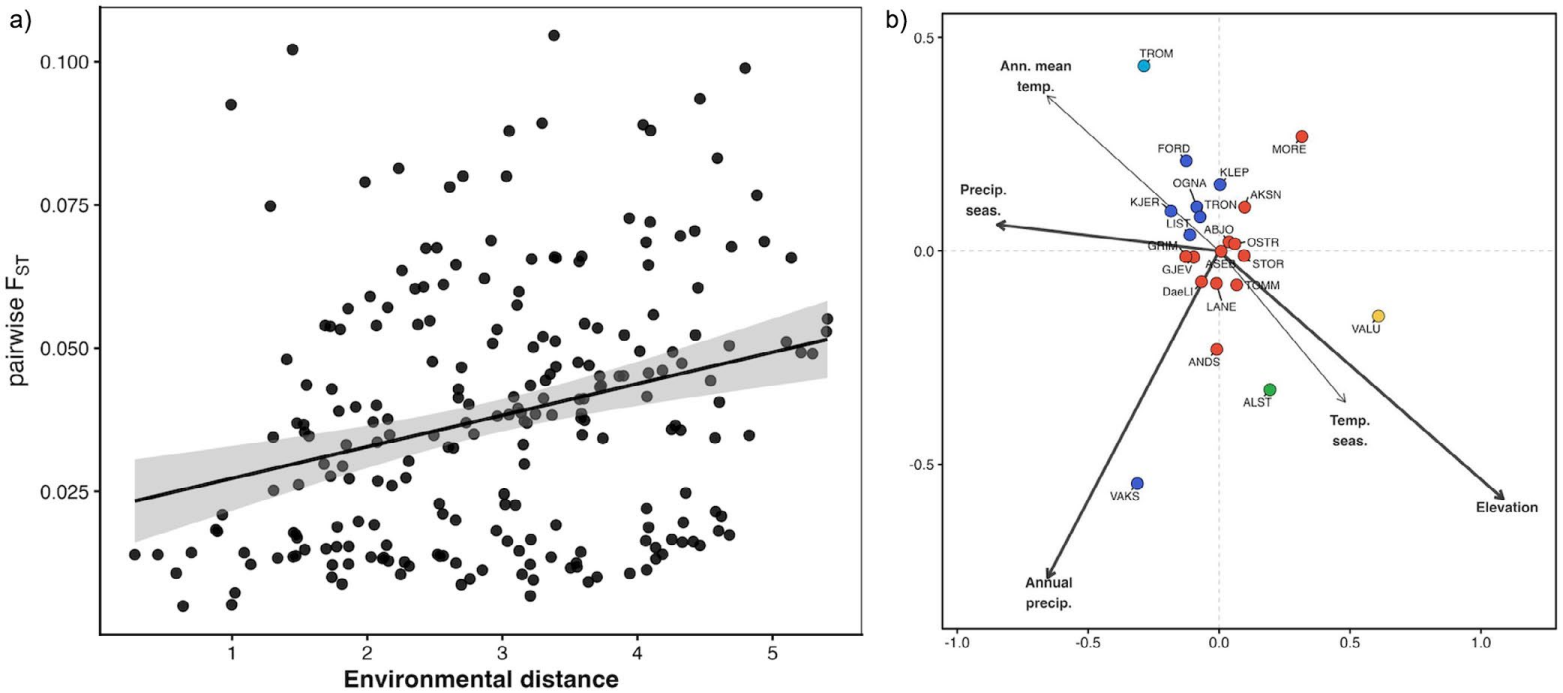
Isolation by Environment (IBE) in *A. millefolium* . (a) Pairwise genetic differentiation (*FST*) increases significantly with environmental distance. The solid line and shaded area show the linear regression and its 95% confidence interval. (b) Partial distance‐based redundancy analysis (dbRDA) shows the effect of environmental variables on genetic differentiation, controlling for geographic distance. Population centroids are colored according to their predominant ADMIXTURE ancestry cluster (*K* = 5), and vectors show the direction and strength of environmental gradients driving genomic variation.

Within‐population genetic diversity showed moderate variation among populations within each species (Table 3). In *S. dioica* , mean within‐population *H*_*O*_ and *H*_*E*_ were 0.248 and 0.221, respectively, with the highest values observed in VOSS (*H*_*O*_ = 0.278, *H*_*E*_ = 0.238) and the lowest in LEIR (*H*_*O*_ = 0.216) and BRON (*H*_*E*_ = 0.195). In *A. millefolium* , mean within‐population heterozygosity was lower (*H*_*O*_ = 0.226, *H*_*E*_ = 0.206), with the lowest values in TRON (*H*_*O*_ = 0.212) and TROM (*H*_*E*_ = 0.190), and the highest in AKSN (*H*_*O*_ = 0.239, *H*_*E*_ = 0.215). At the genome‐wide scale, mean heterozygosity across all filtered SNPs was substantially lower in both species, as expected for ddRAD‐seq data, *H*_*O*_/*H*_*E*_ values of 0.054/0.081 in *S. dioica* and 0.038/0.060 in *A. millefolium* (Table 3).

**TABLE 3:** Within‐population estimates of genome‐wide genetic diversity for Norwegian populations of *S. dioica* and *A. millefolium* based on ddRAD‐seq data. Observed heterozygosity (*H*_*O*_), expected heterozygosity (*H*_*E*_), and inbreeding coefficient (*F*_*IS*_).

Species	PopID	*n*	*H*_*O*_	*H*_*E*_	*F*_*IS*_
*Silene dioica*	ALST	30	0.247	0.227	−0.055
	FINN	40	0.251	0.218	−0.096
	BRON	20	0.223	0.195	−0.087
	GJER	20	0.273	0.228	−0.126
	GRIM	20	0.237	0.215	−0.055
	JARO	20	0.242	0.225	−0.044
	KLEP	20	0.251	0.228	−0.069
	LARV	20	0.228	0.218	−0.035
	LEIR	18	0.216	0.208	−0.021
	LISL	20	0.243	0.223	−0.047
	NORD	20	0.224	0.216	−0.035
	OGNA	18	0.229	0.221	−0.022
	OSTR	20	0.264	0.230	−0.093
	TJON	20	0.262	0.212	−0.175
	TROM	20	0.257	0.212	−0.126
	TRON	20	0.269	0.231	−0.097
	VOSS	20	0.278	0.238	−0.113
*Achillea millefolium*	ABJO	20	0.234	0.213	−0.056
	AKSN	18	0.239	0.215	−0.065
	ALST	19	0.232	0.198	−0.112
	ANDS	19	0.230	0.211	−0.052
	ASEB	20	0.223	0.207	−0.039
	DaeLI	20	0.232	0.211	−0.057
	FORD	20	0.232	0.206	−0.074
	GJEV	20	0.226	0.209	−0.042
	GRIM	20	0.236	0.215	−0.055
	KJER	19	0.219	0.203	−0.040
	KLEP	20	0.218	0.207	−0.023
	LANE	20	0.225	0.204	−0.055
	LIST	20	0.228	0.211	−0.041
	MORE	20	0.213	0.195	−0.050
	OGNA	20	0.219	0.207	−0.026
	OSSL	20	0.228	0.211	−0.041
	OSTR	20	0.230	0.208	−0.059
	STOR	20	0.232	0.210	−0.058
	TOMM	20	0.231	0.213	−0.048
	TROM	20	0.215	0.190	−0.078
	TRON	20	0.212	0.199	−0.030
	VAKS	20	0.216	0.195	−0.061
	VALU	20	0.230	0.193	−0.125

## 4 Discussion

### 4.1 Contrasting Spatial Genomic Structure in Two Widespread Grassland Species

Using genome‐wide population genomic data and extensive sampling across Norway's diverse climatic and latitudinal gradients, we show a striking contrast in spatial genetic structuring between *S. dioica* and *A. millefolium* . Despite sharing key biological and ecological characteristics including insect pollination, a perennial life‐history, a broad geographic distribution and frequent sympatric use in restoration, the two species differ greatly in both the scale and magnitude of population genetic structure. *S. dioica* shows spatially coherent and moderate population differentiation, and significant IBD. In contrast, *A. millefolium* exhibits largely continuous genetic variation characterized by weak population differentiation and extensive admixture among populations. Flow cytometry revealed no ploidy variation in either species, with uniform diploid and hexaploid levels detected in *S. dioica* and *A. millefolium*, respectively (Table S4), suggesting that cytotype variation unlikely to explain the observed genomic patterns in both species.

Contrasting patterns of spatial genetic structuring among broadly sympatric plant species have been widely reported and are often associated with differences in life‐history traits, reproductive biology, dispersal mechanisms, and demographic history (Luna et al. 2005; Duminil et al. 2007; Holderegger et al. 2010; Chung et al. 2023). Our findings add to a growing body of evidence suggesting that effective seed sourcing for restoration should be guided by species‐specific seed transfer zones, rather than generalized seed transfer zones (McKay et al. 2005; Broadhurst et al. 2008; Breed et al. 2019; Bucharova et al. 2019). Numerous studies demonstrate that such zones should consider interspecific differences in genetic structure, dispersal mechanisms, and adaptive potential, thereby reducing the risk of maladaptation and genetic erosion (McKay et al. 2005; Broadhurst et al. 2008; Prober et al. 2015; Jørgensen et al. 2016; Breed et al. 2019; Bucharova et al. 2019). Ideally, species‐specific seed transfer zones should be informed by empirical knowledge of population genetic structure and evolutionary dynamics, rather than relying on broadly defined regions or administrative boundaries (e.g., McKay et al. 2005; Broadhurst et al. 2008). Such administrative boundaries often fail to capture biologically meaningful patterns of genetic differentiation and local adaptation, despite being commonly practiced by several countries, including Norway (Cevallos et al. 2020; Nielsen et al. 2021). Integrating genomic data into seed sourcing strategies therefore provides a knowledge‐based foundation for maintaining genetic integrity, enhancing climate resilience, and promoting long‐term restoration success.

### 4.2 Moderate Genetic Structuring and IBD in *S. dioica*

The moderate spatial genetic structuring observed in *S. dioica* is consistently supported across complementary analyses including PCA, ADMIXTURE, AMOVA, and IBD tests. Population differentiation was substantial (mean pairwise *F*_*ST*_ = 0.13), with AMOVA revealing that 7% of the total variation was partitioned among populations. The observed magnitude of genetic differentiation fits well with estimates reported from the detailed population genetic analysis of *S. dioica* from northern Sweden, which demonstrated moderate subpopulation differentiation and limited pollen‐ and seed‐mediated gene flow, even over fine spatial scales (Giles and Goudet 1997). Such levels of differentiation likely reflect the combined effects of obligate outcrossing, limited pollen, and seed dispersal in heterogeneous environments (Loveless and Hamrick 1984; Duminil et al. 2007; Aguirre‐Liguori et al. 2021). Genetic differentiation increases continuously with geographic distance, resulting in a latitudinal gradient of genetic clustering rather than sharp genetic breaks over short spatial distances. This pattern is characteristic of strong IBD, in which limited dispersal interacts with genetic drift to generate gradual spatial divergence (Wright 1943; Slatkin 1993; Rousset 1997). Such patterns suggest that gene flow in *S. dioica* operates primarily at shorter distances, with limited capacity to homogenize populations across larger distances.

Importantly, genetic structuring in *S. dioica* is not only organized along a broad latitudinal gradient but also marked by internal differentiation. ADMIXTURE analyses (*K* = 7) reveal strong divergence of the TJON population and clear genetic separation between the northern populations of Finnmark and Tromsø, which are separated by approximately 235 km despite sharing similar high‐latitude environments. These patterns indicate limited genetic connectivity among populations across broad spatial scales and likely reflect a combined effect of historical colonization and contemporary barriers to gene flow. Following postglacial recolonization, multiple refugial sources contributed to the colonization of the Scandinavian flora, leaving persistent phylogeographic structure across many taxa (Taberlet et al. 1998; Alsos et al. 2005; Eidesen et al. 2007). In *S. dioica* , chloroplast DNA analyses demonstrate geographically structured haplotypes across Scandinavia, reflecting colonization from multiple refugia (Prentice et al. 2008). Although our genome‐wide SNP data represent nuclear variation, the broad spatial structuring observed here agrees with the previous phylogeographic studies. Thus, limited long‐distance seed dispersal following initial colonization, combined with subsequent isolation and genetic drift, likely promoted the present‐day genetic structure, particularly at range margins marked by limited gene flow.

Norway's complex and rugged topography, characterized by mountain ranges, deep fjords, and steep climatic and latitudinal gradients, may have amplified both historical and contemporary constraints on gene flow, leading to moderate genetic differentiation. This interpretation is in agreement with evidence presented in a comparative study of *Arabidopsis lyrata* which demonstrated that populations in mountainous Norway are more strongly differentiated and genetically less diverse than those in flatter regions such as Sweden, supporting the role of topography as limiting factor to gene flow across northern Europe (Falahati‐Anbaran et al. 2014).

The obligate outcrossing and self‐incompatible breeding system of *S. dioica* may further reinforce this pattern by restricting successful reproduction to pollen exchange among nearby individuals, thereby limiting effective gene flow as geographic distance increases over larger spatial scales (Hamrick and Godt 1996; Barrett 2002). Under such conditions, reproduction depends on pollen exchange among nearby individuals, and when pollen and seed dispersal are spatially restricted, genetic differentiation is expected to accumulate gradually with geographic distance (Wright 1943; Rousset 1997). Our genetic diversity estimates revealed consistently higher observed than expected heterozygosity (*H*_*O*_>*H*_*E*_) and uniformly negative *F*_*IS*_ values, indicating an absence of inbreeding. This pattern aligns with the obligate outcrossing and dioecious breeding system of *S. dioica* , which maintains relatively high within‐population heterozygosity while reducing inbreeding (Loveless and Hamrick 1984; Hamrick and Godt 1996; Barrett 2002). Further, moderate population differentiation and significant isolation by distance indicate geographically restricted gene flow, allowing genetic drift to generate divergence among populations despite high local diversity (Wright 1943; Rousset 1997). All these point to a model in which high genetic diversity is maintained within populations, while limited dispersal and historical processes promote gradual spatial genetic structuring across Norway.

### 4.3 Weak Genetic Structuring and Continuous Genetic Variation in *A. millefolium*

Unlike *S. dioica* , *A. millefolium* shows largely continuous genetic variation across Norway, with high levels of admixture and weak population differentiation. Genome‐wide PCA shows that populations from divergent regions form a single continuous cloud of genetic variation and ADMIXTURE analyses similarly reveal extensive ancestry sharing with little geographic structure. Mean pairwise differentiation is low (*F*_*ST*_ = 0.04), with only 2.4% of the total genetic variation attributed to differences among populations. Furthermore, IBD is weak indicating that geographical separation alone does not explain the observed lack of genetic structuring in this species. However, despite this overall weak genetic structure, the significant IBE detected suggests that environmental variation actively shapes the genomic landscape. In particular, annual precipitation emerged as the strongest predictor of genomic differentiation, suggesting environmental contribution to the observed variation. The observed IBE pattern largely matches Norway's regional precipitation gradients reported by Konstali and Sorteberg (2022), indicating that variation in moisture regimes may act as an environmental barrier to gene flow and contribute to the subtle genomic structuring observed within an otherwise highly connected gene pool.

The weak spatial structure observed in *A. millefolium* may also be facilitated by its effective dispersal mechanisms, including wind‐mediated seed dispersal via lightweight achenes and generalist insect pollination, both of which facilitate gene flow and promote genetic homogenization across large spatial scales. In addition to natural dispersal, *A. millefolium* may also have been dispersed unintentionally through historical hay transport or seasonal livestock movements, including long‐distance transfer from rural to urban areas and in large‐scale local harvests of winter feed in uncultivated outland (Austrheim and Eriksson 2001; Svalheim and Sickel 2017), potentially further weakening spatial genetic structures. In polyploid taxa, increased allelic diversity may further reduce the effects of genetic drift, promoting a gradual allele‐frequency gradient rather than discrete genetic breaks associated with strong genetic structuring (Soltis and Soltis 2000; Parisod et al. 2010; Edgeloe et al. 2022). All these processes may explain why ADMIXTURE identifies shallow ancestry components despite the absence of sharply defined geographic clusters.

Within this continuous homogeneous genetic background, the VALU population from the Telemark region represents a localized deviation from the general pattern. This divergent population shows distinct clustering in PCA and ADMIXTURE analyses, despite sharing the same hexaploid cytotype as all other examined populations, making deep evolutionary divergence or cryptic lineage differentiation unlikely. Instead, this pattern is best interpreted as possibly triggered by local demographic or historical processes such as founder effects, reduced local connectivity, or prolonged persistence under limited gene flow, which can generate pronounced genetic differentiation even within an otherwise well‐connected system (Excoffier et al. 2009; Aguirre‐Liguori et al. 2021). Such localized departures are expected in widespread polyploid plant complexes where stochastic demographic processes can generate pronounced local differentiation without disrupting large‐scale connectivity or indicating long‐term isolation (Parisod et al. 2010; Edgeloe et al. 2022). This species reproduces efficiently asexually, and the steep, heavily grazed mountainside at VALU may have favored the now‐dominant genotype by restricting flowering and thereby limiting sexual reproduction at this site. Accordingly, the divergence of VALU does not contradict, but rather fits within, the overall pattern of weak genetic structure observed across Norway.

Although ADMIXTURE cross‐validation identified *K* = 5 as the statistically supported optimal group, the ancestral components at this *K* are largely shared among populations and show little population structuring. In a species characterized by high gene flow and continuous genetic structure, it is likely that the model‐based clustering approach often captures shallow allele‐frequency gradients, historical admixture or local demographic effects instead of clearly delineating evolutionary units (Pritchard et al. 2000; Lawson et al. 2018). Consequently, despite the statistical support of multiple ancestral components, all analyses indicate that *A. millefolium* populations in Norway show a largely continuous gene pool with only shallow structure.

The lack of strong genetic structuring is likely associated with the biology and evolutionary history of *A. millefolium* which represents a polyploid species complex characterized by a wide ecological tolerance, high dispersal capacity, and extensive gene flow across its range. The broad genomic connectivity observed here agrees with the species' uniform cytogenetic background in the region. While Central European populations of the *A. millefolium* comprise diverse ploidy levels, large‐scale surveys demonstrate that Northern European and Fennoscandian, in particular, the Norwegian lineages are uniformly hexaploid (2n = 6× = 54; Guo et al. 2012; Baltisberger and Widmer 2016). Our flow cytometry analyses further confirmed a uniform hexaploid cytotype across all screened populations, demonstrating that the observed genomic structure is not attributable to ploidy differences. This pattern is also evidenced in its broad evolutionary history; phylogenetic analysis based on nuclear and plastid markers across Central Europe, Asia, and North America indicate rapid diversification and weak geographic structuring across its range, consistent with recent expansion and a lack of long‐term isolation (Guo et al. 2012). Similarly, microsatellite data across the Iberian Peninsula show that most genetic variation was contained within populations, with little differentiation among populations or across ploidy levels, also indicating recent expansion and ongoing gene flow (Lopez‐Vinyallonga et al. 2015).

### 4.4 Species‐Specific Seed Transfer Zones Inferred From Genomic Data

The proposed genomic seed sourcing zones (Table 4) are derived primarily from patterns of genome‐wide neutral population structure and should therefore be interpreted with caution, as they are intended to describe genetic compatibility among seed sources rather than to define adaptive provenance boundaries and optimal source populations. Recent advances in restoration and landscape genomics have shown that integrating neutral population genomic analyses with genotype‐environment association approaches provides complementary insights into environmentally associated genomic variation and adaptive differentiation relevant to ecological restoration and improving seed sourcing strategies (Supple et al. 2018; Breed et al. 2019; Omondi et al. 2024). Comprehensive restoration strategies should integrate these genomic insights with experiments explicitly testing local adaptation across Norway's pronounced climatic and latitudinal gradients. To address this, reciprocal transplant experiments are currently underway across six climatically contrasting localities spanning strong variation in temperature regimes and photoperiod, with ongoing phenotyping of key life‐history and phenological traits. These experiments will allow direct assessment of local adaptation, including potential responses to daylength variation, which is likely to represent an important selective driver across Norway's extreme latitudinal gradient.

**TABLE 4:** Proposed genomic seed‐sourcing zones and their correspondence to current Norwegian seed‐sourcing regions for *Silene dioica* and *Achillea millefolium* . Source populations and full administrative region names are provided in Table 1.

Species	Current seed‐sourcing regions	Proposed genomic zone
*Silene dioica*	Regions 1, 4, 6	Southeastern inland
*Silene dioica*	Regions 2, 3b	Southwestern coastal
*Silene dioica*	Regions 7, 8	Central Norway
*Silene dioica*	Region 7	TJON‐local
*Silene dioica*	Region 9	Western Arctic
*Silene dioica*	Region 9	Eastern Arctic
*Achillea millefolium*	Regions 1, 6, Region 7 (MORE, STOR), and Region 8 (TOMM)	South‐central eastern
*Achillea millefolium*	Regions 2, 3b, Region 7 (AKSN, TRON)	Western coastal
*Achillea millefolium*	Region 9	Northern
*Achillea millefolium*	Region 4	VALU‐local
*Achillea millefolium*	Region 8	ALST‐local

In *S. dioica* , the strength and consistency of spatial genomic structure provide a robust basis for delineating seed transfer zones. The far‐northern Finnmark population (FINN) forms the most strongly differentiated cluster in the dataset, showing minimal admixture and high divergence from all other populations. These results support recognizing FINN as a discrete Eastern Arctic genomic seed transfer zone. In contrast, despite its close geographic proximity (~235 km) and similarly high‐latitude environments, the Tromsø population (TROM) does not cluster with the Finnmark population. Instead, it forms a distinct northern group with a unique ancestral component and limited connectivity, supporting its consideration as a separate Western Arctic genomic seed transfer zone. A similar pattern of divergence between Finnmark‐West and Finnmark‐East has been reported in *Agrostis mertensii* in Norway (Jørgensen et al. 2016), suggesting that such structuring may reflect a broader biogeographic process rather than a species‐specific pattern.

Moving southward, populations from the central Norway region (TRON, TOMM, ALST, and BRON) cluster together and show low differentiation and can therefore be treated as a single Central Norway genomic seed transfer zone. In contrast, the TJON population is a highly divergent and localized genetic unit, likely shaped by a combination of historical isolation and genetic drift. As such, given its strong genetic differentiation, TJON should therefore be treated as a local provenance unit (TJON‐local zone), with transfer of seed originating from TJON restricted to this population. However, its genetic distinctiveness alone does not establish that TJON is an appropriate seed source for restoration in surrounding areas, nor does it define the geographic extent of this local provenance unit. Although there is no known history of restoration seeding or deliberate seed introduction at the sampled locality, a historical introduction from a genetically divergent source population cannot be entirely excluded. Additional sampling of populations surrounding TJON would therefore be required to determine the geographic extent and origin of this distinct genetic group and to refine seed‐sourcing recommendations. For genetically structured species such as *S. dioica* , propagation strategies should therefore prioritize local or within‐zone sourcing, with limited admixture to avoid disrupting co‐adapted gene pools. Populations from the southern west coast (KLEP, OGNA, LEIR, GJER, and VOSS) reveal broadly identical admixture patterns with shared dominant ancestry components, supporting their designation as a Southwestern coastal genomic seed transfer zone, despite VOSS occupying a more geographically intermediate and admixed position. In eastern and southeastern Norway, populations including LARV, LISL, GRIM, JARO, NORD, and OSTR also share a dominant ancestry component with varying degrees of admixture, representing a distinct Southeastern inland genomic seed transfer zone. Given the obligate outcrossing and self‐incompatibility of *S. dioica* , seed collection should therefore prioritize multiple robust populations within each genomic zone to maintain genetic diversity while avoiding transfers across major genomic discontinuities. The correspondence between these inferred genomic zones and the current seed‐sourcing regions is presented in Table 4.

In *A. millefolium* , genome‐wide analyses indicate high connectivity and weak population differentiation across Norway. Principal component analyses show extensive overlap among populations; pairwise differentiation is low across most comparisons, and IBD is weak, together indicating that historical gene flow has operated across broader spatial scales. At the same time, ADMIXTURE analyses reveal geographically structured ancestry gradients, with eastern and southeastern populations dominated by one ancestry component, western coastal populations dominated by another, and additional components increasing in frequency toward northern regions. Although these ancestry components are widely shared and admixed, they define three broad genomic regions corresponding to the South‐central eastern, Western coastal, and Northern genomic seed‐sourcing zones, alongside two local zones (VALU and ALST) identified in this study (Table 4). This regional pattern is consistent with the significant IBE detected in this species, in which annual precipitation emerged as the strongest environmental predictor of genomic differentiation and broadly matches Norway's major regional precipitation gradients (Konstali and Sorteberg 2022).

From a seed‐sourcing perspective, these genomic patterns provide a robust framework for defining genomic seed transfer zones. A South‐central eastern zone encompasses populations from the southeastern and inland regions of Norway (DaeLI, GJEV, GRIM, ABJO, ASEB, OSSL, OSTR, MORE, STOR, and TOMM). In contrast, populations from the western coast (KLEP, LIST, OGNA, FORD, KJER, VAKS, TRON, and AKSN) largely share similar genetic composition and can therefore be regarded as a Western coastal genomic seed transfer zone. The northern populations (ANDs, LANE, and TROM) likewise form a coherent Northern genomic seed transfer zone. These recommendations reflect broad patterns of genomic similarity rather than distinct genetic boundaries and should therefore be interpreted as flexible genomic zones. In contrast to this broadly connected pattern, the VALU population to a large degree and the ALST population to a lesser extent show a distinct genomic signature across multiple analyses and are therefore best regarded as separate local zones (VALU‐local and ALST‐local zones; Table 4). Because these inferences are based on a single sampled population from each area, additional sampling across the adjacent localities will be required to determine whether VALU and ALST represent broader genetic units or localized genetic outliers. Given the extensive gene flow, weak genomic differentiation, and uniform hexaploid cytotype observed across *A. millefolium* populations, maintaining genetic diversity within these broad genomic seed‐sourcing zones is likely to be a greater consideration than the risk of outbreeding depression, which is generally uncommon among genetically connected plant populations (Frankham et al. 2011; Bucharova et al. 2019). Accordingly, restoration efforts should prioritize maintaining genetic diversity by sourcing seeds from multiple populations within each genomic zone while avoiding transfers across the major genomic discontinuities identified in this study.

Comparison of the inferred genomic zones with the current seed‐sourcing regions (Table 4) reveals substantial mismatches in how well existing administrative regions reflect biologically meaningful population structure. In both species, several existing seed‐sourcing regions group into broader genomic zones. For example, Regions 1, 4, and 6 for *S. dioica* formed a single Southeastern inland genomic zone, while Regions 1, 6, and part of Regions 7 and 8 for *A. millefolium* merged into a South‐central eastern genomic zone, suggesting that these regions are genetically more similar than the current administrative boundaries imply. There were, however, several populations that appeared to require management at a much smaller, local scale, including the distinct TJON population within Region 7 in *S. dioica* , and the isolated VALU and ALST populations in *A. millefolium* . Overall, the current administrative regions capture some broad genetic patterns but fail to represent the full extent of species‐specific genomic structure revealed here. These comparisons identify where existing seed‐sourcing regions could be merged and where locally distinct populations may require separate management.

More broadly, our findings demonstrate that species sharing similar ecology and geographical distributions can differ markedly in the scale and strength of genomic structure, underscoring that effective restoration cannot rely on uniform seed sourcing strategies, even across sympatric taxa. Incorporating genomic data into seed‐sourcing strategies is therefore crucial for tailoring species‐specific seed transfer zones that take into consideration dispersal capacity, life‐history traits, and evolutionary history. Future integration of genome‐wide population structure with adaptive genomic analyses and experimental evaluation of local adaptation will provide an increasingly robust framework for developing climate‐resilient seed‐sourcing strategies that preserve both genetic integrity and adaptive potential under environmental change.

## Author Contributions

**Abel Gizaw:** conceptualization (equal), data curation (equal), formal analysis (equal), investigation (equal), methodology (equal), software (lead), visualization (equal), writing – original draft (lead). **Hans Martin Hanslin:** conceptualization (equal), data curation (equal), funding acquisition (equal), investigation (equal), methodology (equal), project administration (equal), supervision (equal), writing – review and editing (equal). **Trygve S. Aamlid:** conceptualization (equal), funding acquisition (equal), investigation (equal), project administration (equal), supervision (equal), validation (equal), writing – review and editing (equal). **Håkon Borch:** conceptualization (equal), funding acquisition (equal), investigation (equal), project administration (equal), resources (equal), supervision (equal), writing – review and editing (equal). **Hana Šípková:** methodology (equal), software (equal), writing – review and editing (equal). **Carl Gunnar Fossdal:** conceptualization (equal), funding acquisition (equal), investigation (equal), methodology (equal), resources (equal), supervision (equal), writing – review and editing (equal).

## Funding

This work was funded by the Norwegian Institute of Bioeconomy Research (NIBIO), project number 53316.

## Disclosure

Benefit‐Sharing Statement: Plant material was collected in Norway in accordance with national regulations. This study complies with the principles of the Nagoya Protocol on Access and Benefit‐Sharing. No genetic resources subject to access restrictions or associated traditional knowledge were used. The genomic data generated in this study are made openly available to support transparency, reproducibility, and the advancement of ecological restoration and conservation genetics research.

## Conflicts of Interest

The authors declare no conflicts of interest.

## Supporting information


**Figure S1:** Genetic structure of *A. millefolium* across Norway without the divergent VALU population.
**Figure S2:** Isolation‐by‐distance patterns based on Rousset's a for *Silene dioica* (a) and *Achillea millefolium* (b). Pairwise genetic differentiation is plotted against the logarithm of geographic distance between populations. Solid lines represent fitted linear regressions and shaded areas indicate 95% confidence intervals.
**Figure S3:** (a) Assignment of ancestry coefficients from ADMIXTURE analysis for *Silene dioica* (*K* = 2 up to *K* = 7). Each vertical bar represents an individual plant, with colors indicating the proportional assignment to different ancestral genetic clusters. Population labels along the x‐axis correspond to the sampling localities listed in Table 1. (b) Assignment of ancestry coefficients from ADMIXTURE analysis for *Achillea millefolium* (*K* = 2 up to *K* = 5). Each vertical bar represents an individual plant, with colors indicating the proportional assignment to different ancestral genetic clusters. Population labels along the x‐axis correspond to the sampling localities listed in Table 1.
**Table S1:** Sample information and sequencing statistics for *Silene dioica* and *Achillea millefolium* . For each individual, the table reports species identity, sample ID, population ID (PopID), percentage of loci recovered (coverage %), and mean sequencing depth following ddRAD‐seq filtering.
**Table S2:** ADMIXTURE cross‐validation (CV) error across *K* = 2–10 for *Achillea millefolium* and *Silene dioica* .
**Table S3:** Results of linear models testing isolation by distance in *A. millefolium* and *S. dioica* . Pairwise genetic differentiation and Rousset's a were regressed against geographic distance among population pairs. Reported statistics summarize model fit and significance.
**Table S4:** Flow cytometric screening of ploidy levels in *Silene dioica* and *Achillea millefolium* . Mean fluorescence values of the internal standard and sample peaks, corresponding coefficients of variation (CV), calculated fluorescence index (sample/standard), and inferred ploidy levels are shown for each analyzed individual.
**Table S5:** Marginal permutation test results for the partial dbRDA showing the degrees of freedom (Df), sums of squares, *F*‐statistics, and permutation‐based *p* values for each environmental predictor in *Achillea millefolium* . Statistically significant results (*p* < 0.05) are shown in bold.

## Data Availability

Raw sequence and filtered SNP datasets used to produce the result in this study are publicly available in the Figshare repository at: https://figshare.com/s/4f0bf2d06022840f9523.

## References

[ece374474-bib-0001] Aguirre‐Liguori, J. A. , S. Ramírez‐Barahona , and B. S. Gaut . 2021. “The Evolutionary Genomics of Species' Responses to Climate Change.” Nature Ecology & Evolution 5, no. 10: 1350–1360. 10.1038/s41559-021-01526-9 34373621

[ece374474-bib-0002] Alexander, D. H. , J. Novembre , and K. Lange . 2009. “Fast Model‐Based Estimation of Ancestry in Unrelated Individuals.” Genome Research 19, no. 9: 1655–1664. 10.1101/gr.094052.109 PMC2752134 19648217

[ece374474-bib-0003] Alsos, I. G. , T. Engelskjon , L. Gielly , P. Taberlet , and C. Brochmann . 2005. “Impact of Ice Ages on Circumpolar Molecular Diversity: Insights From an Ecological Key Species.” Molecular Ecology 14: 2739–2753. 10.1111/j.1365-294X.2005.02621.x. 16029475

[ece374474-bib-0004] Austrheim, G. , and O. Eriksson . 2001. “Plant Species Diversity and Grazing in the Scandinavian Mountains– Patterns and Processes at Different Spatial Scales.” Ecography 24, no. 6: 683–695. 10.1111/j.1600-0587.2001.tb00530.x.

[ece374474-bib-0080] Baltisberger, M. , and A. Widmer . 2016. “Chromosome Numbers and Karyotypes Within the Genus Achillea (Asteraceae: *Anthemideae*).” Willdenowia 46, no. 1: 121–135.

[ece374474-bib-0005] Barrett, S. C. H. 2002. “The Evolution of Plant Sexual Diversity.” Nature Reviews Genetics 3, no. 4: 274–284. 10.1038/nrg776. 11967552

[ece374474-bib-0006] Blischak, P. D. , L. S. Kubatko , and A. D. Wolfe . 2018. “SNP Genotyping and Parameter Estimation in Polyploids Using Low‐Coverage Sequencing Data.” Bioinformatics 34, no. 3: 407–415. 10.1093/bioinformatics/btx587. 29028881

[ece374474-bib-0007] Bower, A. D. , J. B. S. Clair , and V. Erickson . 2014. “Generalized Provisional Seed Zones for Native Plants.” Ecological Applications 24, no. 5: 913–919. 10.1890/13-0285.1. 25154085

[ece374474-bib-0008] Breed, M. F. , P. A. Harrison , A. Bischoff , et al. 2018. “Priority Actions to Improve Provenance Decision‐Making.” Bioscience 68, no. 7: 510–516.

[ece374474-bib-0009] Breed, M. F. , P. A. Harrison , and C. Blyth . 2019. “The Potential of Genomics for Restoring Ecosystems and Biodiversity.” Nature Reviews Genetics 20: 615–628. 10.1038/s41576-019-0152-0. 31300751

[ece374474-bib-0010] Broadhurst, L. M. , A. Lowe , and D. J. Coates . 2008. “Seed Supply for Broadscale Restoration: Maximizing Evolutionary Potential.” Evolutionary Applications 1: 587–597. 10.1111/j.1752-4571.2008.00045.x. PMC3352390 25567799

[ece374474-bib-0011] Bucharova, A. , O. Bossdorf , N. Hölzel , J. Kollmann , R. Prasse , and W. Durka . 2019. “Mix and Match: Regional Admixture Provenancing Strikes a Balance Among Different Seed‐Sourcing Strategies for Ecological Restoration.” Conservation Genetics 20, no. 1: 7–17.

[ece374474-bib-0012] Cevallos, D. , Á. Bede‐Fazekas , E. Tanács , et al. 2020. “Seed Transfer Zones Based on Environmental Variables Better Reflect Variability in Vegetation Than Administrative Units: Evidence From Hungary.” Restoration Ecology 28, no. 4: 911–918.

[ece374474-bib-0013] Chang, C. C. , C. C. Chow , L. C. Tellier , S. Vattikuti , S. M. Purcell , and J. J. Lee . 2015. “Second‐Generation PLINK: Rising to the Challenge of Larger and Richer Datasets.” GigaScience 4: 7. 10.1186/s13742-015-0047-8. PMC4342193 25722852

[ece374474-bib-0014] Chung, M. Y. , J. Merilä , J. Li , et al. 2023. “Neutral and Adaptive Genetic Diversity in Plants: An Overview.” Frontiers in Ecology and Evolution 11: 1116814.

[ece374474-bib-0015] Danecek, P. , A. Auton , and G. Abecasis . 2011. “The Variant Call Format and VCFtools.” Bioinformatics 27: 2156–2158. 10.1093/bioinformatics/btr330. PMC3137218 21653522

[ece374474-bib-0016] Danecek, P. , J. K. Bonfield , and J. Liddle . 2021. “Twelve Years of SAMtools and BCFtools.” GigaScience 10: giab008. 10.1093/gigascience/giab008. PMC7931819 33590861

[ece374474-bib-0017] Doležel, J. , J. Greilhuber , and J. Suda . 2007. “Estimation of Nuclear DNA Content in Plants Using Flow Cytometry.” Nature Protocols 2, no. 9: 2233–2244. 10.1038/nprot.2007.310 17853881

[ece374474-bib-0018] Duminil, J. , S. Fineschi , A. Hampe , et al. 2007. “Can Population Genetic Structure Be Predicted From Life‐History Traits?” American Naturalist 169, no. 5: 662–672. 10.1086/513490 17427136

[ece374474-bib-0019] Durka, W. , S. G. Michalski , J. Höfner , et al. 2025. “Assessment of Genetic Diversity Among Seed Transfer Zones for Multiple Grassland Plant Species Across Germany.” Basic and Applied Ecology 84: 50–60.

[ece374474-bib-0020] Edgeloe, J. M. , A. A. Severn‐Ellis , P. E. Bayer , et al. 2022. “Extensive Polyploid Clonality Was a Successful Strategy for Seagrass to Expand Into a Newly Submerged Environment.” Proceedings of the Royal Society B 289, no. 1976: 20220538. 10.1098/rspb.2022.0538 PMC9156900 35642363

[ece374474-bib-0021] Edmands, S. 2007. “Between a Rock and a Hard Place: Evaluating the Relative Risks of Inbreeding and Outbreeding for Conservation and Management.” Molecular Ecology 16, no. 3: 463–475. 10.1111/j.1365-294X.2006.03148.x. 17257106

[ece374474-bib-0022] Eidesen, P. B. , I. G. Alsos , M. Popp , Ø. Stensrud , J. Suda , and C. Brochmann . 2007. “Nuclear vs. Plastid Data: Complex Pleistocene History of a Circumpolar Key Species.” Molecular Ecology 16: 3902–3925. 10.1111/j.1365-294X.2007.03425.x 17850553

[ece374474-bib-0023] Excoffier, L. , M. Foll , and R. J. Petit . 2009. “Genetic Consequences of Range Expansions.” Annual Review of Ecology, Evolution, and Systematics 40: 481–501. 10.1146/annurev.ecolsys.39.110707.173414.

[ece374474-bib-0024] Falahati‐Anbaran, M. , S. Lundemo , and H. K. Stenøien . 2014. “Genetic Consequences of Climatic Gradients in *Arabidopsis lyrata* .” Molecular Ecology 23: 3031–3044. 10.1111/mec.12784.

[ece374474-bib-0025] Frankham, R. , J. D. Ballou , M. D. B. Eldridge , et al. 2011. “Predicting the Probability of Outbreeding Depression.” Conservation Biology 25, no. 3: 465–475. 10.1111/j.1523-1739.2011.01662.x. 21486369

[ece374474-bib-0026] Fremout, T. 2021. “Seed Sourcing Strategies for Ecological Restoration: A Review of Genomic Approaches.” Biological Conservation 256: 109034. 10.1016/j.biocon.2021.109034.

[ece374474-bib-0027] Giles, B. E. , and J. Goudet . 1997. “Genetic Differentiation in *Silene dioica* Metapopulations: Estimation of Spatiotemporal Effects in a Successional Plant Species.” American Naturalist 149, no. 3: 507–526.

[ece374474-bib-0028] Guo, Y.‐P. , F. Ehrendorfer , and R. Samuel . 2012. “Phylogeny and Systematics of *Achillea* Inferred From Nuclear ITS and Plastid trnL‐F DNA Sequences.” Taxon 61, no. 4: 813–830.

[ece374474-bib-0029] Hamrick, J. L. , and M. J. W. Godt . 1996. “Effects of Life History Traits on Genetic Diversity in Plant Species.” Philosophical Transactions of the Royal Society B 351, no. 1345: 1291–1298. 10.1098/rstb.1996.0112.

[ece374474-bib-0030] Hijmans, R. J. , E. Williams , C. Vennes , and M. R. J. Hijmans . 2017. “Package ‘Geosphere’.” Spherical Trigonometry 1, no. 7: 1–45.

[ece374474-bib-0031] Holderegger, R. , D. Buehler , F. Gugerli , and S. Manel . 2010. “Landscape Genetics of Plants.” Trends in Plant Science 15, no. 12: 675–683. 10.1016/j.tplants.2010.09.002. 20940103

[ece374474-bib-0032] Hörandl, E. , and O. Paun . 2007. “Patterns and Sources of Genetic Diversity in Apomictic Plants: Implications for Evolutionary Potentials.” In Apomixis: Evolution, Mechanisms and Perspectives, 169–194. ARG Gantner Verlag KG.

[ece374474-bib-0033] Hufford, K. M. , and S. J. Mazer . 2003. “Plant Ecotypes: Genetic Differentiation in the Age of Ecological Restoration.” Trends in Ecology & Evolution 18, no. 3: 147–155. 10.1016/S0169-5347(03)00002-8.

[ece374474-bib-0034] IPBES . 2019. “Global Assessment Report on Biodiversity and Ecosystem Services of the Intergovernmental Science‐Policy Platform on Biodiversity and Ecosystem Services. IPBES Secretariat.” 1148.

[ece374474-bib-0035] Jenkins, T. L. 2024. “Mapmixture: An R Package and Web App for Spatial Visualisation of Admixture and Population Structure.” Molecular Ecology Resources 24, no. 4: e13943. 10.1111/1755-0998.13943 38390660

[ece374474-bib-0036] Jørgensen, M. H. , A. Elameen , N. Hofman , S. Klemsdal , S. Malaval , and S. Fjellheim . 2016. “What's the Meaning of Local? Using Molecular Markers to Define Seed Transfer Zones for Ecological Restoration in Norway.” Evolutionary Applications 9, no. 5: 673–684. 10.1111/eva.12378 PMC4869409 27247618

[ece374474-bib-0081] Karger, D. N. , O. Conrad , J. Böhner , et al. 2017. “Climatologies at High Resolution for the Earth's Land Surface Areas.” Scientific Data 4, no. 1: 170122. 10.1038/sdata.2017.122. PMC5584396 28872642

[ece374474-bib-0037] Kolde, R. 2025. “pheatmap: Pretty Heatmaps (R Package Version 1.0.13).” https://CRAN.R‐project.org/package=pheatmap.

[ece374474-bib-0038] Konstali, K. , and A. Sorteberg . 2022. “Why Has Precipitation Increased in the Last 120 Years in Norway?” Journal of Geophysical Research: Atmospheres 127, no. 15: e2021JD036234.

[ece374474-bib-0039] Krauss, S. L. , E. A. Sinclair , J. D. Bussell , and R. J. Hobbs . 2013. “An Ecological Genetic Delineation of Local Seed‐Source Provenance for Ecological Restoration.” Ecology and Evolution 3, no. 7: 2138–2149. 10.1002/ece3.595. PMC3728953 23919158

[ece374474-bib-0040] Lawson, D. J. , L. van Dorp , and D. Falush . 2018. “A Tutorial on How Not to Over‐Interpret STRUCTURE and ADMIXTURE Bar Plots.” Nature Communications 9, no. 1: 3258. 10.1038/s41467-018-05257-7. PMC6092366 30108219

[ece374474-bib-0041] Linck, E. , and C. J. Battey . 2019. “Minor Allele Frequency Thresholds Strongly Affect Population Structure Inference With Genomic Data Sets.” Molecular Ecology Resources 19: 639–647. 10.1111/1755-0998.12995. 30659755

[ece374474-bib-0042] Lopez‐Vinyallonga, S. , I. Soriano , A. Susanna , J. M. Montserra , C. Roquet , and N. Garcia‐Jacas . 2015. “The Polyploid Series of the *Achillea millefolium* Aggregate in the Iberian Peninsula Investigated Using Microsatellites.” PLoS One 10, no. 6: e0129861. 10.1371/journal.pone.0129861 PMC4474640 26091537

[ece374474-bib-0043] Loveless, M. D. , and J. L. Hamrick . 1984. “Ecological Determinants of Genetic Structure in Plant Populations.” Annual Review of Ecology and Systematics 15: 65–95.

[ece374474-bib-0044] Lowe, W. H. , R. P. Kovach , and F. W. Allendorf . 2017. “Population Genetics and Demography Unite Ecology and Evolution.” Trends in Ecology & Evolution 32, no. 2: 141–152. 10.1016/j.tree.2016.12.002. 28089120

[ece374474-bib-0045] Luna, R. , B. K. Epperson , and K. Oyama . 2005. “Spatial Genetic Structure of Two Sympatric Neotropical Palms With Contrasting Life Histories.” Heredity 95, no. 4: 298–305. 10.1038/sj.hdy.6800655. 15815713

[ece374474-bib-0046] Mantel, N. 1967. “The Detection of Disease Clustering and a Generalized Regression Approach.” Cancer Research 27: 209–220. 6018555

[ece374474-bib-0047] McKay, J. K. , C. E. Christian , S. Harrison , and K. J. Rice . 2005. “How Local Is Local? A Review of Practical and Conceptual Issues in the Genetics of Restoration.” Restoration Ecology 13, no. 3: 432–440. 10.1111/j.1526-100X.2005.00058.x.

[ece374474-bib-0048] Meirmans, P. G. , S. Liu , and P. H. van Tienderen . 2018. “The Analysis of Polyploid Genetic Data.” Journal of Heredity 109, no. 3: 283–296. 10.1093/jhered/esy006. 29385510

[ece374474-bib-0049] Naturmangfoldloven . 2009. “Lov om forvaltning av naturens mangfold (LOV‐2009‐06‐19‐100). Lovdata.” https://lovdata.no/dokument/NL/lov/2009‐06‐19‐100.

[ece374474-bib-0050] Newbold, T. , L. N. Hudson , A. P. Arnell , et al. 2016. “Has Land Use Pushed Terrestrial Biodiversity Beyond the Planetary Boundary? A Global Assessment.” Science 353, no. 6296: 288–291. 10.1126/science.aaf2201. 27418509

[ece374474-bib-0051] Newbold, T. , L. N. Hudson , S. L. L. Hill , et al. 2015. “Global Effects of Land Use on Local Terrestrial Biodiversity.” Nature 520: 45–50. 10.1038/nature14324. 25832402

[ece374474-bib-0052] NIBIO . 2026. “Artsrike blomsterfrøblandinger og tilhørende naturgrasblandinger for ti soner.” https://www.nibio.no/tema/landskap/nibio‐landvik‐norsk‐kompetansesenter‐for‐blomstereng‐og‐naturfro/artsrike‐blomsterfroblandinger‐og‐tilhorende‐naturgrasblandinger‐for‐ti‐soner?locationfilter=true.

[ece374474-bib-0053] Nielsen, A. , I. E. Måren , L. Rosef , et al. 2021. “Assessment of Possible Adverse Consequences for Biodiversity When Planting Vascular Plants Outside Their Natural Range in Norway. VKM Report:15. Norwegian Scientific Committee for Food and Environment (VKM).”

[ece374474-bib-0054] Oksanen, J. , F. G. Blanchet , M. Friendly , et al. 2017. “vegan: Community Ecology Package.” R package version 2.4‐4.

[ece374474-bib-0055] O'Leary, S. J. , J. B. Puritz , S. C. Willis , C. M. Hollenbeck , and D. S. Portnoy . 2018. “These Aren't the Loci You're Looking for: Principles of Effective SNP Filtering for Molecular Ecologists.” Molecular Ecology 27: 3193–3206. 10.1111/mec.14792. 29987880

[ece374474-bib-0056] Omondi, E. O. , C. Y. Lin , S. M. Huang , et al. 2024. “Landscape Genomics Reveals Genetic Signals of Environmental Adaptation of African Wild Eggplants.” Ecology and Evolution 14, no. 7: e11662. 10.1002/ece3.11662 PMC11232056 38983700

[ece374474-bib-0057] Parisod, C. , R. Holderegger , and C. Brochmann . 2010. “Evolutionary Consequences of Autopolyploidy.” New Phytologist 186, no. 1: 5–17. 10.1111/j.1469-8137.2009.03142.x. 20070540

[ece374474-bib-0058] Patterson, N. , A. L. Price , and D. Reich . 2006. “Population Structure and Eigen Analysis.” PLoS Genetics 2, no. 12: e190. 10.1371/journal.pgen.0020190. PMC1713260 17194218

[ece374474-bib-0059] Peterson, B. K. , J. N. Weber , E. H. Kay , H. S. Fisher , and H. E. Hoekstra . 2012. “Double Digest RADseq: An Inexpensive Method for de Novo SNP Discovery and Genotyping in Model and Non‐Model Species.” PLoS One 7: e37135. 10.1371/journal.pone.0037135. PMC3365034 22675423

[ece374474-bib-0060] Piqueray, J. , V. Gilliaux , V. Decruyenaere , J.‐T. Cornelis , R. Uyttenbroeck , and G. Mahy . 2019. “Management of Grassland‐Like Wildflower Strips Sown on Nutrient‐Rich Arable Soils: The Role of Grass Density and Mowing Regime.” Environmental Management 63: 647–657. 10.1007/s00267-019-01153-y 30868313

[ece374474-bib-0061] Prentice, H. C. , J. U. Malm , and L. Hathaway . 2008. “Chloroplast DNA Variation in the European Herb *Silene dioica* (Red Campion): Postglacial Migration and Interspecific Introgression.” Plant Systematics and Evolution 272, no. 1: 23–37. 10.1007/s00606-007-0629-8.

[ece374474-bib-0062] Pritchard, J. K. , M. Stephens , and P. Donnelly . 2000. “Inference of Population Structure Using Multilocus Genotype Data.” Genetics 155: 945–959. 10.1093/genetics/155.2.945 PMC1461096 10835412

[ece374474-bib-0063] Prober, S. M. , M. Byrne , E. H. McLean , et al. 2015. Climate‐Adjusted Provenancing: A Strategy for Climate‐Resilient Ecological Restoration. Vol. 3, 65. Frontiers Media SA.

[ece374474-bib-0064] Purcell, S. , B. Neale , K. Todd‐Brown , et al. 2007. “PLINK: A Tool Set for Whole‐Genome Association and Population‐Based Linkage Analyses.” American Journal of Human Genetics 81, no. 3: 559–575. 10.1086/519795. PMC1950838 17701901

[ece374474-bib-0065] Pustahija, F. , S. C. Brown , F. Bogunic , et al. 2013. “Small Genomes Dominate in Plants Growing on Serpentine Soils in West Balkans, an Exhaustive Study of 8 Habitats Covering 308 Taxa.” Plant and Soil 373: 427–453. 10.1007/s11104-013-1794-x.

[ece374474-bib-0066] R Core Team . 2023. “R: A Language and Environment for Statistical Computing, Vol. R Foundation for Statistical Computing. City.” http://www.R‐project.org.

[ece374474-bib-0067] Rousset, F. 1997. “Genetic Differentiation and Estimation of Gene Flow From F‐Statistics Under Isolation by Distance.” Genetics 145, no. 4: 1219–1228. 10.1093/genetics/145.4.1219 PMC1207888 9093870

[ece374474-bib-0068] Runfola, D. , A. Anderson , H. Baier , et al. 2020. “geoBoundaries: A Global Database of Political Administrative Boundaries.” PLoS One 15, no. 4: e0231866. 10.1371/journal.pone.0231866 PMC7182183 32330167

[ece374474-bib-0069] Slatkin, M. 1993. “Isolation by Distance in Equilibrium and Non‐Equilibrium Populations.” Evolution 47, no. 1: 264–279. 10.1111/j.1558-5646.1993.tb01215.x 28568097

[ece374474-bib-0070] Šmarda, P. , O. Knápek , A. Březinová , et al. 2019. “Genome Sizes and Genomic Guanine+ Cytosine (GC) Contents of the Czech Vascular Flora With New Estimates for 1700 Species.” Preslia 91, no. 2: 117–142.

[ece374474-bib-0071] Soltis, P. S. , and D. E. Soltis . 2000. “The Role of Genetic and Genomic Attributes in the Success of Polyploids.” Proceedings of the National Academy of Sciences of the United States of America 97: 7051–7057. 10.1073/pnas.97.13.7051. PMC34383 10860970

[ece374474-bib-0072] Suda, J. , and P. Trávníček . 2006. “Reliable DNA Ploidy Determination in Dehydrated Tissues of Vascular Plants by DAPI Flow Cytometry ‐ New Prospects for Plant Research.” Cytometry, Part A 69: 273–280. 10.1002/cyto.a.20253. 16528735

[ece374474-bib-0073] Supple, M. A. , J. G. Bragg , L. M. Broadhurst , et al. 2018. “Landscape Genomic Prediction for Restoration of a *Eucalyptus* Foundation Species Under Climate Change.” eLife 7: e31835. 10.7554/eLife.31835 PMC5951681 29685183

[ece374474-bib-0074] Svalheim, E. , and H. Sickel . 2017. “Seed Dispersal of Semi‐Natural Grassland Plants Through Historical Travel and Use ‐ As a Basis for the Conscious Use of Local and Regional Seed Material Today. NIBIO Report 155/3. Ås: Norwegian Institute of Bioeconomy Research.” https://hdl.handle.net/11250/2498029.

[ece374474-bib-0075] Taberlet, P. , L. Fumagalli , A. G. Wust‐Saucy , and J. F. Cosson . 1998. “Comparative Phylogeography and Postglacial Colonization Routes in Europe.” Molecular Ecology 7: 453–464. 10.1046/j.1365-294x.1998.00289.x. 9628000

[ece374474-bib-0076] Waples, R. S. , and O. Gaggiotti . 2006. “What Is a Population? An Empirical Evaluation of Some Genetic Methods for Identifying the Number of Gene Pools and Their Degree of Connectivity.” Molecular Ecology 15, no. 6: 1419–1439. 10.1111/j.1365-294X.2006.02890.x. 16629801

[ece374474-bib-0077] Weir, B. S. , and C. C. Cockerham . 1984. “Estimating F‐Statistics for the Analysis of Population Structure.” Evolution 38: 1358–1370. 10.1111/j.1558-5646.1984.tb05657.x. 28563791

[ece374474-bib-0078] Wickham, H. 2016. ggplot2: Elegant Graphics for Data Analysis. Springer.

[ece374474-bib-0079] Wright, S. 1943. “Isolation by Distance.” Genetics 28, no. 2: 114–138. 10.1093/genetics/28.2.114 PMC1209196 17247074

